# SLUG and Truncated TAL1 Reduce Glioblastoma Stem Cell Growth Downstream of Notch1 and Define Distinct Vascular Subpopulations in Glioblastoma Multiforme

**DOI:** 10.3390/cancers13215393

**Published:** 2021-10-27

**Authors:** Sophie Guelfi, Béatrice Orsetti, Virginie Deleuze, Valérie Rigau, Luc Bauchet, Hugues Duffau, Bernard Rothhut, Jean-Philippe Hugnot

**Affiliations:** 1 VIB-KU Leuven Center for Cancer Biology, 3000 Leuven, Belgium; 2 Institut des Neurosciences de Montpellier, University of Montpellier (UM), Institut National de la Santé et la Recherche Médicale (INSERM), 34091 Montpellier, France; bernard.rothhut@inserm.fr; 3 Institut de Recherche en Cancérologie de Montpellier, University of Montpellier (UM), INSERM, 34298 Montpellier, France; beatrice.orsetti@inserm.fr; 4 Institut de Génétique Moléculaire de Montpellier, University of Montpellier (UM), Centre National de la Recherche Scientifique (CNRS), 34293 Montpellier, France; virginie.deleuze@igmm.cnrs.fr; 5 Department of Pathology and Oncobiology, Hôpital Gui de Chauliac, 34295 Montpellier, France; v-rigau@chu-montpellier.fr; 6 Institut de Génomique Fonctionelle, University of Montpellier (UM), CNRS, INSERM, 34094 Montpellier, France; l-bauchet@chu-montpellier.fr (L.B.); h-duffau@chu-montpellier.fr (H.D.); 7 Neurosurgery Department, Hôpital Gui de Chauliac, 34295 Montpellier, France; 8 Department of Biology, University of Montpellier (UM), CEDEX 5, 34095 Montpellier, France

**Keywords:** glioblastoma multiforme (GBM), glioblastoma stem cells (GSC), GSC growth, GBM microenvironment, notch signaling, TGF-β signaling, transcription factors, SLUG (SNAI2), TAL1 (SCL), endothelial cells

## Abstract

**Simple Summary:**

Glioblastoma multiforme is the most aggressive form of brain tumor and is still incurable. These neoplasms are particularly difficult to treat efficiently because of their highly heterogeneous and resistant characteristics. Advances in genomics have highlighted the complex molecular landscape of these tumors and the need to further develop effective and targeted therapies for each patient. A specific population of cells with enriched stem cell properties within tumors, i.e., glioblastoma stem cells (GSC), drives this cellular heterogeneity and therapeutical resistance, and thus constitutes an attractive target for the design of innovative treatments. However, the signals driving the maintenance and resistance of these cells are still unclear. We provide new findings regarding the expression of two transcription factors in these cells and directly in glioblastoma patient samples. We show that these proteins downregulate GSC growth and ultimately participate in the progression of gliomas. The forthcoming results will contribute to a better understanding of gliomagenesis.

**Abstract:**

Glioblastomas (GBM) are high-grade brain tumors, containing cells with distinct phenotypes and tumorigenic potentials, notably aggressive and treatment-resistant multipotent glioblastoma stem cells (GSC). The molecular mechanisms controlling GSC plasticity and growth have only partly been elucidated. Contact with endothelial cells and the Notch1 pathway control GSC proliferation and fate. We used three GSC cultures and glioma resections to examine the expression, regulation, and role of two transcription factors, SLUG (SNAI2) and TAL1 (SCL), involved in epithelial to mesenchymal transition (EMT), hematopoiesis, vascular identity, and treatment resistance in various cancers. In vitro, SLUG and a truncated isoform of TAL1 (TAL1-PP22) were strongly upregulated upon Notch1 activation in GSC, together with LMO2, a known cofactor of TAL1, which formed a complex with truncated TAL1. SLUG was also upregulated by TGF-β1 treatment and by co-culture with endothelial cells. In patient samples, the full-length isoform TAL1-PP42 was expressed in all glioma grades. In contrast, SLUG and truncated TAL1 were preferentially overexpressed in GBMs. SLUG and TAL1 are expressed in the tumor microenvironment by perivascular and endothelial cells, respectively, and to a minor extent, by a fraction of epidermal growth factor receptor (EGFR) -amplified GBM cells. Mechanistically, both SLUG and truncated TAL1 reduced GSC growth after their respective overexpression. Collectively, this study provides new evidence for the role of SLUG and TAL1 in regulating GSC plasticity and growth.

## 1. Introduction

Glioblastoma multiforme (GBM) is the deadliest form of brain malignancy, for which there is no effective therapy [1]. The inevitable failure of the currently used standard-of-care protocols results from multiple factors specific to this disease [2]. As reflected by its denomination, GBM is a highly heterogeneous neoplasm. Seminal genomic studies revealed an intertumoral heterogeneity between patients and defined distinct molecular subtypes, namely proneural, mesenchymal, classical, and neural subtypes [3]. Furthermore, the intratumoral heterogeneity of GBMs was also demonstrated by the coexistence of multiple tumor subclones within the same tumor [4]. Investigating this heterogeneity is thus a necessary step towards effective and targeted therapies for patients [5]. Moreover, GBMs are complex ecosystems, in which tumor cells rapidly adapt to the surrounding microenvironment, namely, neural, immune, stromal, and vascular cells that actively participate in GBM progression and therapeutical resistance [6,7].

The identification of stem-like cells with tumorigenic and radio/chemoresistant properties adds an extra layer of complexity [8]. These cells, termed glioblastoma stem cells (GSCs), share key characteristics with neural stem cells and are the main drivers of GBM intratumoral heterogeneity [9]. GSCs are fully integrated within their microenvironmental landscape, in specifically-defined niches [10]. Notably, by providing Notch1 ligands, endothelial cells exert a tight control on GSC proliferation and differentiation within the perivascular niche [11,12,13]. Given their crucial contribution to tumor progression and resistance, a better understanding of the signals driving their phenotype and plasticity is necessary in order to consider GSCs as attractive targets for effective therapies [14].

Transcription factors are central drivers of GSC self-renewal, proliferation, and differentiation downstream of dysregulated developmental signaling pathways, including Hippo/Yap [15], TGF-β1 [16], and Notch1 [17]. Recently, we have shown that Notch1 pathway activation in GSCs blocks their proliferation, inhibits ASCL1, OLIG2, and SOX2 expression, and promotes their differentiation into pericyte-like cells, both in vitro and in vivo [18]. A similar role for Notch1 activation was also observed in isocitrate dehydrogenase 1 (IDH1) mutant diffuse low-grade gliomas [19]. These studies provided us with a specific molecular signature and a non-exhaustive list of transcription factors that potentially mediate the Notch1 downstream effects. Among these candidates, we observed that *SLUG (SNAI2)* and *TAL1 (SCL)* RNAs were the most upregulated following Notch1 activation in GSCs. SLUG (or SNAI2) is a zinc finger transcriptional repressor belonging to the Snail superfamily, and is a master regulator of epithelial cell motility and migration during embryonic and tumoral epithelial-to-mesenchymal transition processes (EMT) [20,21,22,23]. Importantly, only a few reports have questioned the contribution of SLUG during gliomagenesis, so far linking its expression to glioma grade [24], mesenchymal GBM subtypes [25] and therapeutic recurrence [26]. TAL1 (or SCL) is a class II bHLH transcription factor first described as a master oncogenic driver of T-cell acute lymphoblastic leukemias (T-ALL) [27,28]. During embryogenesis, it is a key determinant of hematopoietic [29,30], erythroid [31], and endothelial lineages [32,33], as well as controlling spinal cord neurogenesis [34,35,36] and astrogenesis [37] in the developing central nervous system (CNS). In the adult, TAL1 is active during pathological angiogenesis [38,39,40] and vascular remodeling [41], and has been rarely studied in solid tumors. In CNS tumors, it is expressed in hemangioblastomas [42] and could act as an oncogene in glioma [43].

To date, the expression, regulation, and contribution of SLUG and TAL1 transcription factors during gliomagenesis have been poorly documented, especially using current models for gliomas that maximize relevance to this pathology. Here we addressed this issue by using three GSC cultures isolated from patients which we previously characterized for cardinal GSC properties, i.e., their self-renewal, multipotency, and tumorigenic potential [44,45]. We studied whether SLUG and TAL1 affected GSC growth and how they were regulated in these cells by Notch1 and TGF-β1 signaling, as well as by the presence of endothelial cells. Finally, we examined in detail the expression of TAL1 and SLUG in the tumor microenvironment and tumoral cells. For this purpose, we used diffuse low-grade and high-grade patient resections to perform immunofluorescence with specific lineage markers and also established a technique combining fluorescence in situ hybridization (FISH) with immunofluorescence. Collectively, these results expand our previous knowledge and understanding of the downstream effectors of Notch1 pathway in GSC and the role SLUG and TAL1 in these cells.

## 2. Materials and Methods

### 2.1. Cell Culture

Glioblastoma stem-like cells (GSC) were previously isolated from human glioblastoma resections and fully characterized to meet GSC requirements; i.e., multipotency, neural stem cell marker expression, and tumor initiation capacity upon orthotopic xenografts. Gb4 and Gb7 cells were used for all experiments [18,44]; Gb21 cells were additionally used to confirm specific results [45]. GSCs were used under 5 passages and maintained as neurospheres in a proliferation media consisting of DMEM/F12 1:1 (Thermo Fisher, Illkirch, France) supplemented with N2 (Thermo Fisher), D-glucose (0.6%, Sigma-Aldrich Chimie, St. Quentin Fallavier, France), L-glutamine (2 mM, Thermo Fisher), B27 w/o vitamin A (Thermo Fisher), EGF (20 ng/mL, Peprotech, Neuilly-Sur-Seine, France), FGF2 (10 ng/mL, Peprotech), and heparin (2 µg/mL, Sigma-Aldrich Chimie). For immunofluorescence (IF) staining, GSCs were also plated in adherent proliferating conditions, using poly-D-Lysine (PDL) (25 µg/mL, Sigma-Aldrich Chimie) and Laminin (2 µg/cm^2^, Sigma-Aldrich Chimie) coated culture supports, in the same GSC proliferation media. For differentiation, GSCs were cultured in adherent conditions (PDL-Laminin, Sigma-Aldrich Chimie) in a differentiation media consisting of GSC proliferation media without heparin, EGF, and FGF2 growth factors, and supplemented with 0.5% fetal bovine serum (Thermo Fisher). Treatments with control solution (Dymethylsulfoxyde, DMSO) or TGF-β1 (2 ng/mL, Peprotech) were performed in each of these culture conditions, and SLUG induction was measured after 5 days. Single donor human umbilical vein endothelial cells (HUVEC) (PromoCell, Heidelberg, Germany) were cultured in Endothelial Cell Growth Medium 2 (PromoCell) on gelatin coated vessel (0.1%, Sigma-Aldrich Chimie). Primary mesenchymal stem cells MSC103, used as control cells for immunofluorescence, were derived from healthy bone marrow donor and were obtained from EFS (Etablissement Francais du sang, cell collection n° DC-2008-686 in collaboration with Dr. Vignais (IGF, Montpellier)). Regarding co-cultures, 40,000 GSCs (Gb4, Gb7, and Gb21) were plated on top of subconfluent HUVECs in 24-well coverslips coated with gelatin (0.1%, Sigma-Aldrich Chimie), and grown for 72 h until fixation and subsequent staining. The control condition included 40,000 GSCs cultured alone in HUVEC media (PromoCell) on gelatin coated plates. In order to discriminate GSCs from HUVECs, GSCs were transduced with an IRES-YFP lentivirus prior to co-culture (see section below).

### 2.2. Lentiviral Transductions

Control IRES-YFP or NICD-IRES-YFP lentiviruses (gifts from Dr Sutton’s lab, Yale, New Haven, CT, USA) were transduced in proliferating GSCs (Gb4, Gb7, and Gb21) to activate the Notch1 intracellular axis (multiplicity of infection MOI 1:6) [18]. GSCs were collected 5 days post-transduction for immunofluorescence and western blots (WB)/co-immunoprecipitation assays. For overexpression studies, human SNAI2 (SLUG), human TAL1-PP22, and control (luciferase) lentiviral vectors were designed with VectorBuilder GmbH (Neu-Isenburg, Germany). For *SNAI2 (SLUG)*, the human reference sequence NM_003068.4 from NCBI (GenBank) was used. For the TAL1-PP22 isoform, a custom sequence was designed using the reference sequence NM_001290406.2 from NCBI (GenBank). Sequences also integrated an eGFP expression cassette and were cloned into 3rd generation lentiviral vectors. Viruses were produced within the Vector Platform of Montpellier and transduced in proliferating GSCs (Gb4 and Gb7) using an optimized MOI 1:10. Cells were collected 5 days post-transduction for cell growth quantifications and WB analyses.

### 2.3. Human Samples and Histology

Human resections from non-tumoral cortex, IDH1-mutant grade II and III gliomas (referred to as diffuse low-grade gliomas (DLGG)), and glioblastomas (GBM) were obtained from the Biological Resource Bank of the Gui de Chauliac University Hospital of Montpellier, with patient consent and in accordance with the hospital Institutional Review Board (IRB-MTP_2021_03_202100779, IRB Montpellier hospital Accreditation number: 198711). Glioma diagnosis was assessed by a neuropathologist, Pr. Rigau, using World Health Organization (WHO) 2016 criteria that included: detection of IDH1 mutation, 1p19q deletion, loss of ATRX staining, and quantifying p53, Ki67, EGFR stainings [1]. Resections were numbered according to their date of reception; their detailed description and use are listed in Tables S1 and S2. Following surgical resection, samples were kept on ice and further subdivided for protein extractions and histology. For histology, samples were fixed for 2 h at 4 °C in 4% paraformaldehyde solution (Sigma-Aldrich Chimie) (pH 7.0), followed by cryopreservation at 4 °C in gradually concentrated sucrose solutions (Sigma-Aldrich Chimie) (7,5%, 15%, and 30%). Samples were then embedded in Tissue Tek OCT (Sakura Finetek, Torrance, CA, USA) and frozen in −80 °C isopentane using a SnapFrost80 apparatus (Excilone, Elancourt France). Blocks were cut using a Leica 2800E cryostat (Leica Microsystemes, Nanterre, France); and 14 µm sections on Superfrost Plus slides (Thermo Fisher) were kept at −80 °C before subsequent staining.

### 2.4. Immunohistochemistry and Immunofluorescence

Immunohistochemistry (IHC) was performed on 6 µm cryosections of samples using a Bond Polymer Refine Detection Kit (Leica Microsystemes) for peroxidase stainings [46]. IF stainings were performed using previously validated methods [18]. Briefly, permeabilization and blocking were performed with 0.1% Triton X-100 (Sigma-Aldrich Chimie) and 10% donkey serum (Sigma-Aldrich Chimie). Primary antibodies listed in Table S3 were incubated overnight at 4 °C. Secondary Alexa-488 or 594 conjugated antibodies (Jackson ImmunoResearch, Cambridgeshire, UK) were incubated 1 h at room temperature (RT). DAPI (4′,6-diamidino-2-phenylindole) was used for nuclei counterstaining. For IF of cultured cells, coverslips were fixed with 4% paraformaldehyde (Sigma-Aldrich Chimie) for 20 min at RT, and similar methods were used for IF staining on sections. Images were acquired with a Zeiss AxioImager Z2/Apotome epifluorescence microscope (Zeiss, Paris, France); and were analyzed independently by two investigators (S.G. and J.P.H.) using Zen Blue (Zeiss) and Image J software (National Institute of Mental Health, Bethesda, MD, USA). For quantification of IHC images, a minimum of 500 total cells were counted across multiple slides per sample. For IF quantification of cultured GSCs, a minimum of 150 YFP^+^ cells were counted for both NICD transductions and coculture experiments. For IF quantification of GBM resections, a minimum of 100 SLUG^+^ or TAL1^+^ cells were analyzed across multiple slides for each sample and each combination of markers.

### 2.5. Immunofluorescence Followed by Fluorescence In Situ Hybridization (IF-FISH)

A custom method (sequential IF-FISH) was designed to assess EGFR amplification of SLUG^+^ and TAL1^+^ cells in GBM cryosections (GBM#23, #24, #26). First, IF for SLUG or TAL1 was performed as previously described on 6 µm cryosections, using DAPI and Alexa-594 conjugated secondary antibodies for imaging (Jackson ImmunoResearch, Cambridgeshire, UK). Positive cell positions were mapped with a Zeiss AxioImager Z2/Apotome (Zeiss) and saved using Zen Blue (Zeiss). Then, slides were processed for fluorescence in situ hybridization (FISH). Briefly, after pretreatment and co-denaturation at 85 °C, slides were hybridized overnight at 37 °C in a humidified chamber with 3 μL of EGFR 7p11.2/SE7 dual FISH probe (Leica Microsystemes) solubilized in hybridization buffer. Stringent washes (55 °C) were performed to remove unspecific signals, and slides were mounted with DAPI for imaging. The hybridization signals of the EGFR probe coupled to PlatinumBright550 (red) and the Control SE7 probe (Satellite enumeration for Chromosome 7) coupled to PlatinumBright 495 (green) were measured on previously mapped cells using the same settings on the microscope and Zen Blue. Only cells with a clear *EGFR* locus signal in red and a clear signal for *SE7* satellite probe in green were considered for quantification. A cell was considered not amplified when 2 *EGFR* copies (red) and 2 *SE7* copies (green) were clearly identified. A cell was considered amplified when 3 *EGFR* copies (red) or more were identified. Images were analyzed, scored for EGFR locus amplification, and processed for publication using Image J.

### 2.6. Measures of Cell Growth

To assess the effects of SLUG and TAL1-PP22 overexpression on GSC growth, 15,000 transduced Gb4 and Gb7 cells were dissociated, sorted for GFP positivity using a BD FACSAria III, and seeded as proliferating neurospheres in 24-well plates (*n* = 5 wells per conditions). After 5 days of growth, GSCs were dissociated with trypsin directly in the wells (0.5% final) and counted with an automated Z2 Coulter Cell Counter (Beckman Coulter, Villepinte, France). Cell growth measurements were repeated in 3 independent experiments for each cultures using the same protocol.

### 2.7. Western Blots and Co-Immunoprecipitation

Total proteins from human samples and cultured cells were extracted using previously described methods [18,46]. Protein concentration was determined using a Pierce BCA Protein Assay (Thermo Fisher). If not specified otherwise, 30 μg of whole cell lysate was separated by SDS-PAGE using 4–15% precast Protean TGX gels (BioRad, Marnes-la-Coquette, France) and transferred on 0.2 µm PVDF membranes (BioRad). Membranes were blocked in Li-Cor PBS blocking buffer (Li-Cor, Bad Homburg, Germany) for 1 h at room temperature and further incubated with primary antibodies overnight at 4 °C (Table S3). Following TBS-T washes, membranes were incubated for 1 h at room temperature with fluorescently labelled IRDye 680 and 800 secondary antibodies (Li-Cor) and imaged with an Odyssey CLx Imaging System (Li-Cor) and Image Studio software (Li-Cor). Image J was used for image processing and β-actin was used as a loading control in all experiments, unless specified otherwise. Band signal intensities were normalized both with β-actin and control conditions signals. The number of independent repeats (*n*) are indicated for each assay in the main figure legends. When applicable, quantifications and statistics are presented in Figure S9. Original Western blot images can be found at Figures S10–S14. For co-immunoprecipitation, a Pierce Co-IP kit was used, according to the manufacturer’s recommendations (Thermo Fisher). Briefly, after pre-clearing steps, whole cell lysates (WCL) were incubated overnight at 4 °C with 2 µg of TAL1 goat antibody or control IgG (see Table S3), then precipitated with 30 µL of agarose protein A/G beads. Following several washes, precipitated proteins were separated using SDS-PAGE to detect LMO2 protein.

### 2.8. Bioinformatics and Statistical Analyses

Analyses of RNA expression profiles of *SLUG (SNAI2)* and *TAL1* in human gliomas datasets were performed using Gliovis Data Visualization Tools for Brain Tumor Datasets (http://gliovis.bioinfo.cnio.es/, accessed on 9 January 2019). Normalized gene expression datasets from REMBRANDT and TCGA_GBMLGG databases were downloaded, replotted, and analyzed [47]. For single-cell RNA-seq analyses of cell type expression for *SLUG (SNAI2)* and *TAL1* in GBMs, data and plots available from www.gbmseq.org, (accessed on 8 January 2020) were directly integrated in the figure with the consent of Dr. Darmanis and Dr. Gephart [48]. For the expression profiles of *SLUG (SNAI2)* and *TAL1* in different GSC culture subtypes, boxplots from the Human Glioma Cell Culture (HGCC) biobank (www.hggc.se, accessed on 6 January 2021) were directly used in the figure and datasets were downloaded for statistical analyses. All statistical analyses of datasets were performed with GraphPad Prism 8 (San Diego, CA, USA), and notably consisted in ordinary one-way ANOVA with multiple comparisons between the mean of each group and Tukey’s correction. If not mentioned otherwise, experimental data are shown as means +/− standard error of the mean (SEM), and are representative of 3 independent experiments. Statistical differences for IF quantifications and cell counts were measured using the Mann–Whitney rank sum test. If different, specific statistical tests are mentioned in the figure legends. Significances are ****, *p* < 0.0001 ***, *p* < 0.001; **, *p* < 0.01; *, *p* < 0.05.

## 3. Results

### 3.1. SLUG and TAL1 Are Inducible in Cultured GSCs

**Figure 1:**
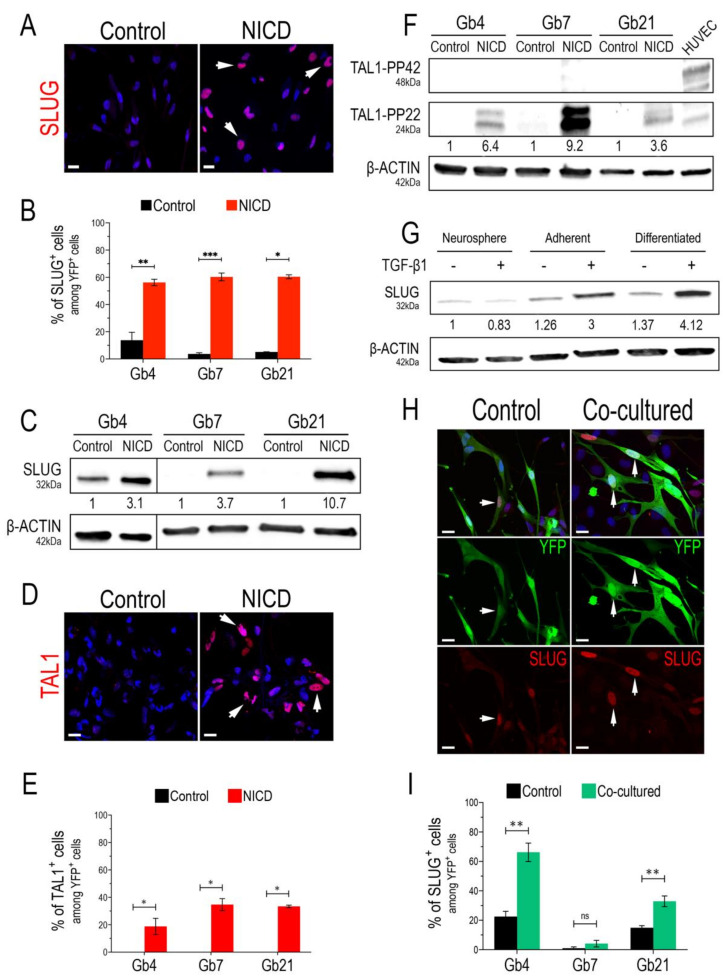
SLUG and TAL1 expression in cultured GSCs. (**A**,**D**) Representative IF images showing SLUG upregulation in Gb4 (**A**) and TAL1 induction (**D**) in Gb7 upon the transduction of the Notch1 IntraCellular Domain (NICD) in proliferating adherent conditions (PDL/Laminin). Arrowheads indicate positive cells; scales 20 µm. (**B**,**E**) Quantification of SLUG^+^/TAL1^+^ cells following Notch1 activation in Gb4, Gb7, and Gb21 cells. (**C**,**F**) Representative WB images and quantifications showing SLUG upregulation (**C**) and TAL1-PP22 induction (**F**) in Gb4, Gb7, and Gb21 cells upon NICD transduction (*n* = 3, Figure S9A,B). (**C**) The original image was modified so that the Gb4 lanes appear on the left. (**G**) SLUG expression and upregulation in Gb4 cells using different culture methods; including proliferating neurospheres, adherent proliferating, and adherent differentiating conditions; with (+) or without (−) TGB-β1 treatment (2 ng/mL). Cells were harvested after 5 days of culture and/or treatment. Proliferating neurosphere conditions without TGB-β1 treatment were used as the basal condition for quantifications (*n* = 3, Figure S9C). (**H**) Representative IF images of SLUG upregulation in Gb4 cells upon co-culture with HUVEC endothelial cells. Cells were cocultured for 72 h and transduced with a YFP lentivirus in order to discriminate them from HUVEC cells. Arrowheads indicate YFP^+^ Gb4 GSCs. scales 20 µm. (**I**) Quantification of YFP^+^SLUG^+^ Gb4, Gb7, and Gb21 cells after co-culture with HUVEC cells (72 h). (**B**,**E**,**I**) Statistical analyses using rank Mann–Whitney tests, *n* = 3, ≥150 YFP^+^ cells quantified per experiment, ***, *p* < 0.001; **, *p* < 0.01; *, *p* ≤ 0.05, n.s. = not significant.

We started our study by confirming the bona fide expression of SLUG and TAL1 transcription factors upon Notch1 activation, using three previously characterized GSC cultures (Gb4, Gb7, and Gb21) [44,45] and validated SLUG and TAL1 antibodies (Figure S1). By lentiviral transduction of GSCs with either control (IRES-YFP) or an activated form of Notch1 (NICD-YFP, Notch1 IntraCellular Domain) in proliferating conditions (i.e., in the presence of growth factors), we first observed a strong overexpression of SLUG in all cultures and its nuclear expression in at least 50% of transduced GSCs (Figure 1A,B and Figure S2A). This strong upregulation was also confirmed by Western blot (WB) analyses, showing a minimal three-fold induction in Gb4 and a maximal 10-fold induction in Gb21 (Figure 1C). Regarding TAL1, we observed its induction in all NICD-transduced GSCs and its nuclear expression in at least 20% of transduced GSCs (Figure 1D,E and Figure S2B). Since distinct isoforms of TAL1 have been previously described during embryonic hematopoiesis [49,50], we further examined the expression of these isoforms in our NICD-transduced GSCs using WB analysis. Interestingly, while HUVECs mainly expressed full-length TAL1, namely the TAL1-PP42 isoform (48kDa), a shorter and truncated 24kDa isoform, namely TAL1-PP22, was solely induced in all GSCs (Figure 1F).

Our lentivirus-based approach to overexpress NICD mimics a constitutive and forced Notch1 activation that might not reflect physiological conditions within tumors. Consequently, we examined whether SLUG and TAL1 could be physiologically inducible in GSCs. We hypothesized that culture conditions and inducing differentiation could influence Notch1 activation in GSCs, potentially triggering a downstream upregulation of both SLUG and TAL1. Thus, we tested the effects of adherent and differentiating culture conditions, and assessed SLUG and TAL1 expression in GSCs. Interestingly, we detected a subset of native SLUG^+^ GSCs (approximately 15%) in the Gb4 line and, overall, a basal expression in these cells; while very few native SLUG^+^ cells were observed in Gb7 and Gb21 cells (Figure 1B). This was confirmed in protein extracts, with no detectable SLUG in Gb7 and Gb21, in contrast to the Gb4 cells (Figure 1C). GSCs can be cultured as 3D neurospheres or in a 2D adherent condition on PDL/Laminin-coated supports. We then compared SLUG expression in these two culture modes and also when cells were differentiated cells by growth factor removal. We observed a slight increase of SLUG expressionin adherent vs. neurosphere Gb4 cultures, which was further increased upon their differentiation (Figure 1G). No changes in SLUG expression levels were detected in Gb7 and Gb21 cells when modulating these culture conditions (data not shown).

Due to its major functions in EMT, SLUG is directly activated via the crosstalk of important signaling axes, involving components of the TGF-β1 cascade [20]. Thus, in addition to modulating the above-mentioned culture conditions, we treated GSCs with TGF-β1 (2 ng/mL) for 5 days. While no effect of TGF-β1 was observed in Gb4 cells cultured as neurospheres, we observed a stronger increase in SLUG expression when plating Gb4 cells on PDL/Laminin coated supports (3-fold), and upon their differentiation (4-fold) (Figure 1G). In addition, TGF-β1 treatment of Gb4 cells induced morphological changes, especially in the differentiation condition (Figure S3). In contrast, no changes in SLUG expression levels were observed in Gb7 and Gb21 cells upon TGF-β1 treatment in these culture conditions (data not shown). Importantly, neither proliferating adherent conditions nor differentiating conditions nor TGF-β1 treatment could trigger the expression of either TAL1-PP42 or TAL1-PP22 in all GSC cultures (data not shown).

It is now well established that GSCs reside within perivascular niches, in which endothelial cells provide essential cues for their self-renewal and differentiation [13]. In turn, GSCs actively participate in tumoral endothelial cell remodeling and contribute to GBM vascularization mechanisms [7]. This interplay is modulated via signals that mainly include Notch1 ligands, as well as TGF-β1 and BMP cytokines [10]. Given the individual effects of NICD overexpression and TGF-β1 treatment on SLUG and TAL1 induction in GSCs, we next assessed whether a physiological contact with endothelial cells could directly trigger their expression. We, thus, used human umbilical vein endothelial cells (HUVEC) to set up a simple two-dimensional GSC-HUVEC co-culture system, by plating YFP^+^ GSCs on top of HUVEC monolayers and growing the cells together for 3 days in HUVEC media (Figure S3). Upon co-culture and immunofluorescence assays, we observed a significant upregulation of SLUG in both Gb4 and Gb21 cells (Figure 1H,I). The strongest upregulation of SLUG with a subset of 60% YFP^+^SLUG^+^ cells was observed in Gb4, while no significant induction was measured in Gb7 cells (Figure 1H,I). Regarding TAL1, we could not induce its expression in any YFP^+^ co-cultured GSCs. However, and as expected, TAL1 was expressed in co-cultured HUVECs with no obvious differences with the control HUVECs (Figure S4).

Taken together, these results indicate that both SLUG and TAL1 are inducible in cultured GSCs. SLUG expression is regulated by activation of Notch1 and TGF-β1 treatment, by a direct contact with endothelial cells and by modulating culture conditions, with variations observed between our different GSC cell lines. In contrast, the truncated TAL1-PP22 isoform is solely induced downstream of Notch1 activation in all GSC cultures.

### 3.2. SLUG and TAL1 Define Mutually Exclusive Subpopulations of Vascular Cells in GBM Resections

**Figure 2:**
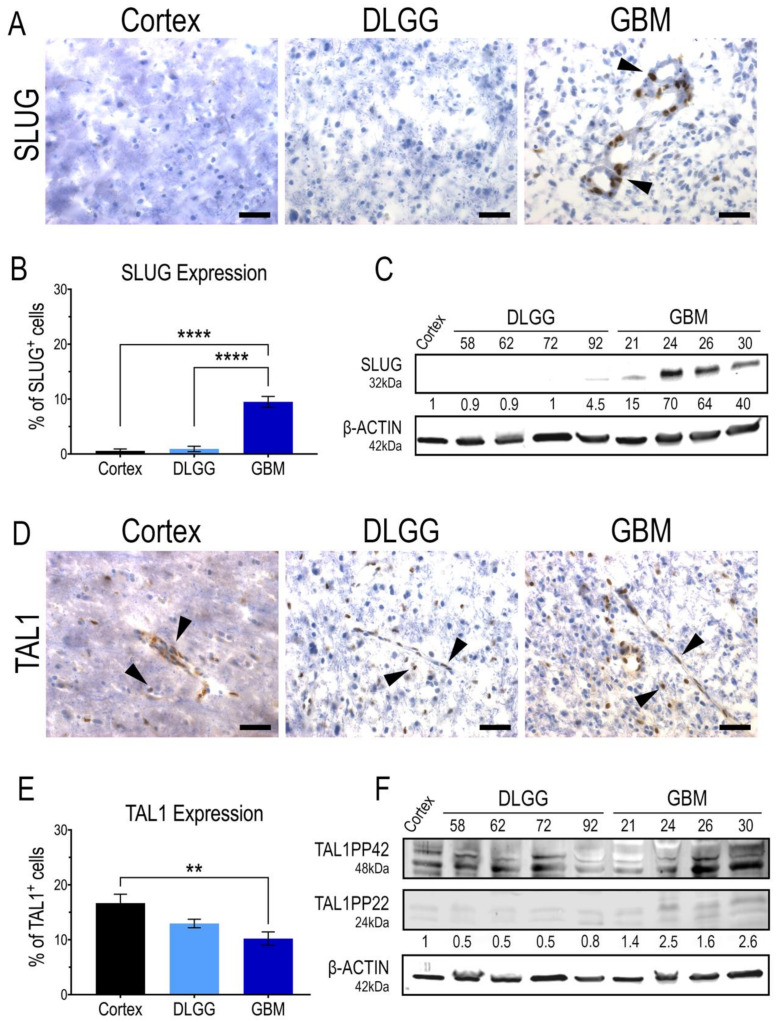
SLUG and TAL1 expression in human glioma samples. (**A**,**D**) Representative immunohistochemistry (IHC) images of SLUG and TAL1 expression in human sample cryosections, including normal cortex (*n* = 1); diffuse low-grade gliomas (DLGG) (*n* = 5) and glioblastomas (GBM) (*n* = 5) (Tables S1 and S2). Nuclear SLUG was mainly observed in GBMs (**A**); while TAL1 expression is both cytoplasmic and nuclear and expressed in all samples (**D**). Arrowheads indicate positive cells; scales 40 µm. (**B**,**E**) Quantifications of SLUG^+^ and TAL1^+^ cells in these samples, ≥500 total cells were counted for each sample, positive cells are represented as % of total cells. Statistical analyses using ordinary one-way ANOVA tests with multiple comparisons between the mean of each group. ****, *p* < 0.0001; **, *p* < 0.01. (**C**,**F**) Whole tumor WB analyses of SLUG and TAL1 expression in human resections, including normal cortex (*n* = 1); DLGG (*n* = 4) and GBM (*n* = 4). A total 50 µg of total protein was loaded. (**F**) TAL1-PP22 isoform is upregulated in GBMs while TAL1-PP42 is expressed in all samples, using a validated antibody detecting all known isoforms of TAL1 (Table S3). Band intensities were normalized with β-actin and are shown as fold changes of the cortex sample.

Considering the expression of SLUG and TAL1 in GSC in vitro, it was important to demonstrate the expression of these two transcription factors in patient resections. Thus, we performed immunohistochemistry analyses on cryosections and Western blots (WB) on tumor samples, using antibodies validated in vitro on GSCs. Our study included a non-tumoral human cortex sample as a control, five diffuse IDH1-mutant grade II and III gliomas and five GBMs. First, we clearly detected a nuclear expression of SLUG in a subset of cells in GBMs. Overall, SLUG expression was significantly higher in GBMs than in the cortex and IDH1-mutant gliomas that did not harbor any, or very few, SLUG^+^ cells (Figure 2A,B). This was confirmed with protein extracts in which SLUG was specifically upregulated in all GBM samples (Figure 2C). Surprisingly, TAL1^+^ cells were present in all samples, the highest nuclear expression being in the human cortex sample, and the lowest being in GBMs (Figure 2D,E). Similar to our WB analyses in GSC cell lines (Figure 1F), the truncated isoform TAL1-PP22 was upregulated in GBMs (Figure 2F). The overall expression pattern of full length TAL1-PP42 was similar to our microscopic observations showing TAL1 expression in all tested samples (Figure 2F). Both SLUG^+^ and TAL1^+^ subpopulations respectively accounted for approximately 10% of total cells across the GBM samples we considered in our study (Figure 2B,E). To extend our observations to a broader number of samples, we analyzed the available genomic databases of human gliomas [47]. Using TCGA and REMBRANDT datasets, we confirmed, at least at the RNA level, that SLUG (SNAI2) expression is significantly higher in GBMs (Figure S5A,B), while TAL1 expression is significantly lower in GBMs (Figure S5C,D).

**Figure 3:**
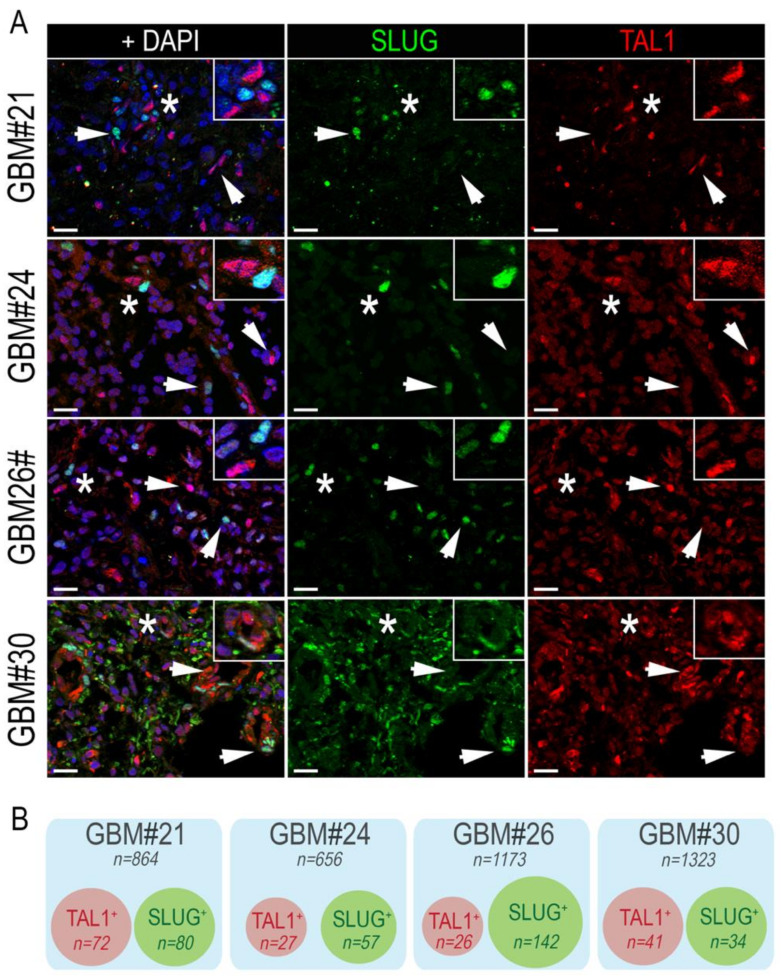
SLUG^+^ and TAL1^+^ cells are mutually exclusive in GBM resections. (**A**) Representative IF images showing the mutually exclusive expression of SLUG and TAL1 in GBM#21, #24, #26, and #30 samples. Arrowheads point to either SLUG^+^ or TAL1^+^ cells. Stars indicate regions where SLUG^+^ and TAL1^+^ cells are in close vicinity and are magnified in upper right-hand corners of images; scales 20 µm. (**B**) Venn diagram representation of SLUG^+^ and TAL1^+^ distinct subpopulations in GBM#21, #24, #26, and #30. Squares represent entire samples, red circles represent TAL1^+^ cells, and green circles represent SLUG^+^ cells; n indicates the number of counted cells for each sample and subsets.

Following the identification of SLUG and TAL1 cell subpopulations directly in GBM samples, we questioned whether these subsets overlapped and examined their co-expression by immunofluorescence, using the same GBM samples. After extensive quantifications, performed in four GBM samples, we found no colocalization of these two transcription factors. However, SLUG^+^ and TAL1^+^ cells were found in close vicinity across all GBM samples (Figure 3A,B).

**Figure 4:**
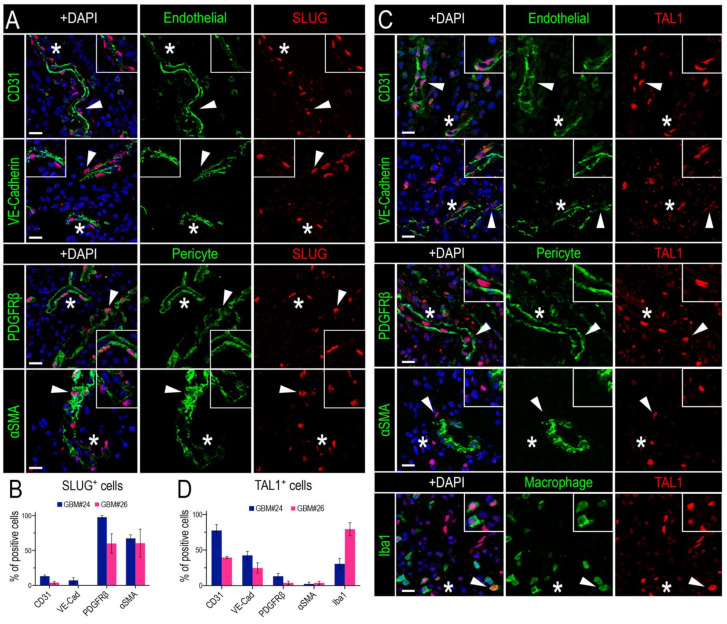
SLUG and TAL1 expression in vascular cells in GBMs. (**A**,**C**) Representative IF images of either SLUG (**A**) or TAL1 (**C**) co-expression with the endothelial markers CD31 and VE-Cadherin, perivascular/pericyte markers PDGFRβ and αSMA, and macrophage/microglial marker Iba1 for TAL1 (**C**) in GBM#24 sample. Arrowheads indicate cells of interest, stars indicate regions of interest magnified in corners of images, altogether showing an expression of SLUG in perivascular cells/pericytes and TAL1 in endothelial cells; scales 20 µm. (**B**,**D**) Co-expression quantifications of CD31, VE-Cadherin, PDGFRβ, αSMA with SLUG^+^ cells (**B**), and the same markers + Iba1 with TAL1^+^ cells (**D**) in GBM#24 and #26. A minimum of 100 SLUG^+^ or TAL1^+^ cells analyzed for each sample and each combination of markers. Double positive cells are represented as % of total SLUG^+^ or TAL1^+^ cells.

To determine the identity of SLUG^+^ and TAL1^+^ cells in GBM, we proceeded with an extensive and thorough characterization using specific combinations of immunofluorescence markers in two GBM biopsies (GBM#24 and #26). First, we noticed that SLUG^+^ cells were often located around the vessels (Figure 2A), suggesting that these cells might be vascular muscle cells, as observed during brain development [51]. Indeed, by performing stainings for SLUG with two well-known smooth muscle cells markers (PDGFRβ and αSMA) and two pan-endothelial markers (CD31 and VE-Cadherin) in two GBM samples, we established that around 50% of SLUG^+^ cells were vascular muscle cells, whereas very few expressed endothelial markers (Figure 4A,B).

Second, by conducting a similar analysis for TAL1^+^ cells, we established that a significant fraction of these cells had an endothelial phenotype, as they expressed CD31 and VE-Cadherin, while very few expressed PDGFRβ and αSMA (Figure 4C,D). In addition, we observed a TAL1^+^ Iba1^+^ subpopulation illustrating the expression of TAL1 in macrophages and microglial cells (Figure 4C,D). Collectively, these results show that SLUG and truncated TAL1-PP22 are specifically upregulated in GBMs and demonstrate the specific and mutually exclusive expression of SLUG and TAL1 in GBM vascular cells.

### 3.3. SLUG and TAL1 Subpopulations Contain Cells of Tumoral Origin in GBM Samples

Our in vitro results indicate that both SLUG and TAL1 can be induced in cultured GSCs. Given the plasticity of these cells and their contribution to GBM heterogeneity and tumorigenicity, we questioned whether the distinct SLUG^+^ and TAL1^+^ subsets identified in GBM resections contained cells of tumoral origin in addition to the vascular and microglial cells we had previously identified (Figure 4).

To validate that SLUG and TAL1 protein are indeed present in a fraction of GBM tumoral cells, we relied on *EGFR* gene amplification as the most common genetic alteration observed in human GBMs [5]. The *EGFR* gene is located in the 7p11.2 locus of chromosome 7, and its amplification using fluorescence in situ hybridization (FISH) is routinely used by pathologists to determine diagnoses of cancer patients.

**Figure 5:**
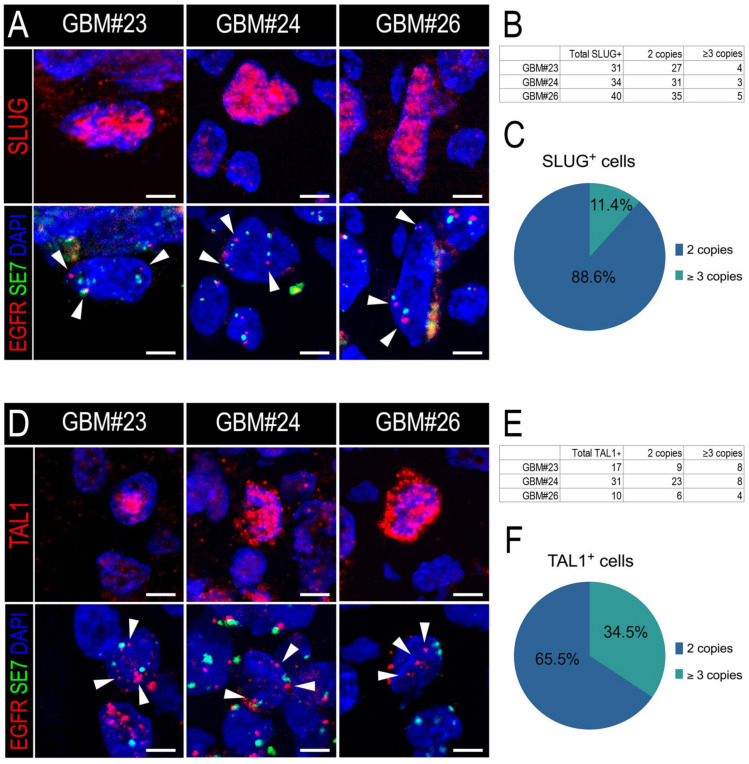
SLUG^+^ and TAL1^+^ cells respectively contain a minor proportion of *EGFR*-amplified cells in GBM resections. (**A**,**D**) Representative images of SLUG^+^ (**A**) and TAL1^+^(**D**) cells amplified for *EGFR* in GBM#23, #24, and #26 using our sequential IF-FISH method. Left panels show IF for SLUG or TAL1 on selected cells, right panels indicate EGFR/SE7 loci on the same cells following hybridization with the dual EGFR/SE7 FISH probe. Amplification was detected by the number of *EGFR* copies (red dots) compared to the number of *SE7* copies (green dots) per nucleus, here serving as a centromeric control for chromosome 7. White arrowheads indicate *EGFR* loci in nuclei. Owing to the applied sequential method and post-treatment for FISH hybridization, nuclei appear slightly different in the right panels; scales 5 µm. DAPI: 4′,6-diamidino-2-phenylindole; EGFR: *EGFR* locus-specific probe; SE7: satellite enumeration probe for chromosome 7. (**B**,**E**) Quantification tables of *EGFR* amplification in SLUG^+^ (**B**) or TAL1^+^ (**E**) cells in each GBM sample. Cells with 3 *EGFR* copies or more were quantified as amplified, cells with 2 *EGFR* copies and 2 *SE7* copies were quantified as non-amplified. (**C**,**F**) Pie graphs showing the average percentage of SLUG^+^ (**C**) or TAL1^+^ (**F**) *EGFR*-amplified versus non-amplified cells across all GBM samples.

Thus, we applied a similar strategy to measure the *EGFR* amplification status of either SLUG^+^ or TAL1^+^ cells. First, we confirmed the *EGFR* amplification of the three GBM resections we considered (GBM#23, #24, and #26) (Figure S6A,B). Furthermore, we developed a custom protocol to combine immunofluorescence of either SLUG or TAL1 with the *EGFR* locus FISH analysis of these samples (IF-FISH). Using a sequential method, we first stained for SLUG or TAL1 and mapped positive cells, then hybridized the *EGFR* fluorescent probe and finally imaged specific cells of interest. In all three GBM biopsies, we detected *EGFR*-amplified SLUG^+^ (Figure 5A) and *EGFR*-amplified TAL1^+^ cells (Figure 5D). We applied a strict scoring method and found that the majority of SLUG^+^ and TAL1^+^ cells were not amplified for the *EGFR* locus and are thus probably not tumoral (Figure 5B,E and Figure S6C,D). However, *EGFR* amplification was clearly observed in 11.4% and 34.5% of scored SLUG^+^ (Figure 5C) and TAL1^+^ cells (Figure 5F), respectively, demonstrating that these two transcription factors can be expressed by some GBM tumoral cells.

Altogether, these results indicate that a minor subset of either SLUG^+^ or TAL1^+^ cells are of tumoral origin, whilst the major sources for these proteins are cells of non-tumoral identity, based on *EGFR* amplification.

### 3.4. SLUG and TAL1-PP22 Independently Control the Growth of GSCs In Vitro

**Figure 6:**
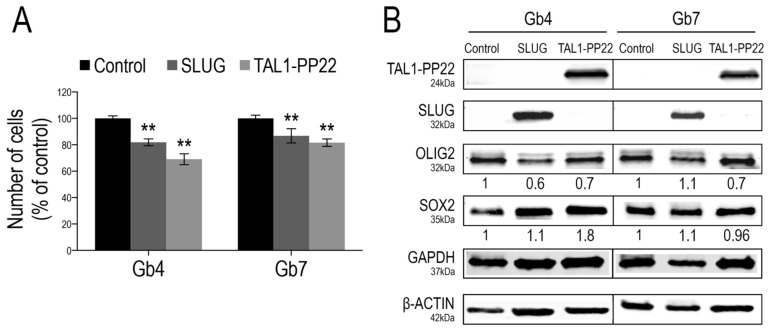
SLUG and TAL1-PP22 overexpression respectively reduce GSC growth. (**A**) Cell counts of Gb4 and Gb7 cells 5 days after transduction of Control, SLUG or TAL1-PP22-overexpressing lentiviruses. Data is shown as a % of the control condition and is representative of 3 independent experiments, *n* = 5 wells per conditions. Statistical analyses using rank Mann–Whitney tests, **, *p* < 0.01. (**B**) WB analyses of Gb4 and Gb7 cells after SLUG or TAL1-PP22 overexpression, showing no mutual modulation of expression and no significant change in OLIG2 and SOX2 expression (*n* = 1). Control for overexpression of SLUG and expression of SOX2 was normalized to β-actin; control for TAL1-PP22 overexpression and expression of OLIG2 was normalized to GAPDH.

To further investigate the functions of SLUG and TAL1 in gliomagenesis, we questioned the consequences of their overexpression in proliferating GSCs using a lentiviral approach. Given the sole induction of truncated TAL1-PP22 in GSCs upon Notch1 activation and its increased expression in GBM resections, we designed constructs expressing this short isoform. First, we validated the overexpression of either SLUG or TAL1-PP22 in Gb4 and Gb7 cells by WB and IF (Figure 6B and Figure S7A,C). Furthermore, using a cell growth assay performed over 5 days, the forced upregulation of SLUG or TAL1-PP22 induced a significant decrease in the cell numbers of both Gb4 and Gb7 cells; the sharpest reduction being observed following truncated TAL1-PP22 overexpression (Figure 6A). However, neither SLUG nor TAL1-PP22 overexpression drastically modulated the expression of pro-proliferative transcriptional regulators OLIG2 and SOX2, which are highly expressed in native proliferating GSCs (Figure 6B and Figure S7B,D). This suggested alternative mechanisms, whereby SLUG and truncated TAL1-PP22 regulate GSC growth, respectively. Interestingly, we also observed that SLUG overexpression did not modify the level of TAL1-PP22 in both Gb4 and Gb7 cells, and vice versa. Thus, SLUG and TAL1 might act independently from one another in cultured GSCs and therefore would define distinct subsets of cells as observed in GBM samples (Figure 6B).

### 3.5. Truncated TAL1-pp22 Interacts with LMO2 upon Notch1 Activation of Cultured GSCs

**Figure 7:**
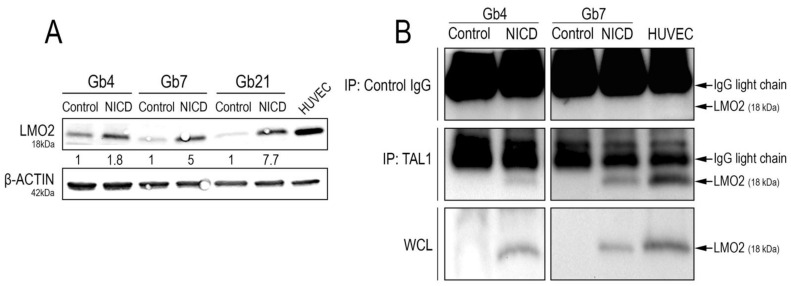
LMO2 upregulation and interaction with TAL1-PP22 upon Notch1 activation of GSCs. (**A**) WB images and quantifications showing LMO2 upregulation in Gb4, Gb7, and Gb21 upon Notch1 activation. HUVEC cells were used as a positive control for LMO2 expression (*n* = 3, Figure S9D). (**B**) TAL1 Co-IP analyses showing an interaction between TAL1-PP22 and LMO2 in Gb4 and Gb7 cells upon NICD transduction. HUVEC cells were used as a positive control. Upper panels show LMO2 WB on control IP using IgG lysates, middle panels show LMO2 WB on TAL1-IP lysates, and lower panels show control LMO2 WB on whole cell lysates (WCL). Gb4 YFP/NICD WCLs and control IgG lysates were loaded on different gels. Given the low molecular weight of LMO2 (18kDa), IgG light chains are apparent on the blot above the LMO2 band. Images are representative of *n* = 2 experiments for Gb4 and *n* = 1 experiment for Gb7.

TAL1 exerts its full transcriptional activity by interacting with defined transcriptional partners and cofactors [52]. Specifically in endothelial cells, TAL1 directly activates endothelial genes via its interaction with the transcriptional cofactor LIM domain only 2 (LMO2) [39,53]. LMO2 was also recently reported as a regulator of tumorigenicity, angiogenesis, and invasion in GSCs and GBMs [54,55]. Remarkably, LMO2 was expressed in native proliferating conditions in all of our GSC cultures (Figure 7A).

Additionally, we observed its strong upregulation upon Notch1 activation, with the highest induction in Gb21 cells (Figure 7A). We next sought to analyze whether Notch1-induced TAL1-PP22 would directly interact with LMO2. Upon TAL1 co-immunoprecipitation assays using NICD-transduced GSCs, we, indeed, confirmed their interaction in both Gb4 and Gb7 cells (Figure 7B).

## 4. Discussion

In this study, we examined the expression and functions of SLUG and TAL1 transcription factors, in gliomas. For relevance to the pathology, we based our work on low passage serum-free GSCs, protein extracts, and sections obtained from patient resections. In vitro, we uncovered the direct upregulation of both SLUG and the short isoform TAL1-PP22 downstream of Notch1 activation in proliferating GSCs, together with LMO2, which interacts with TAL1-PP22. SLUG is also inducible upon GSC differentiation, TGF-β1 treatment, and direct co-culture with endothelial cells. In patient samples, SLUG and TAL1-PP22 were upregulated in grade IV GBMs in mutually exclusive subpopulations of vascular cells, respectively perivascular and endothelial cells, as well as, to a minor extent, in a fraction of EGFR-amplified tumoral cells. Mechanistically, we found that SLUG and TAL1-PP22 independently inhibit GSC growth in vitro. Three main conclusions can be drawn from this work.

First, SLUG and a small isoform of TAL1 are upregulated by Notch1 activation in three patient-derived GSC cell lines in vitro. It is now well-established that the activation of the Notch1 pathway antagonizes glioma proliferation [17]. However, the detailed mechanisms mediating this effect are still elusive. Upon overexpression, we found that SLUG and truncated TAL1-PP22 reduced GSC growth in vitro, and could thus mediate Notch1 anti-proliferative effects in gliomas. Mechanistically, we could not detect an influence of TAL1 and SLUG on the two key glioma transcription factors we explored (OLIG2 and SOX2) so their specific downstream targets remain to be fully identified. Furthermore, SLUG and TAL1-PP22 could also exert their function on GSC growth via regulation of apoptosis, which was not explored in our study.

Second, we found that even without exogenous Notch1 activation, basal expression of SLUG was detected in one GSC cell line (Gb4). This further illustrates the well-known heterogeneity of GBM and derived-GSC lines, which, notably, arises from the different mutational backgrounds. By using the RNA seq database from the Human Glioblastoma Cell Culture resource [56], *SLUG (SNAI2)* was significantly more expressed in GSC cultures with a mesenchymal phenotype (Figure S8A), while *TAL1* expression was not specific to any subtype (Figure S8B). Mesenchymal GSCs typically have a hemizygous deletion of *NF1* gene [3]. Our Gb4 line has a chromosome 7 deletion in q11.2 encompassing the *NF1* gene (unpublished data), suggesting that *SLUG* expression in this line is linked to the mesenchymal profile. Using this Gb4 line, we found that SLUG expression is not only upregulated by the Notch1 pathway, but also by other signals. Indeed, GSC differentiation, TGF-β1 treatment, and co-culture with endothelial cells can upregulate SLUG at the protein level. Endothelial cells highly express ligands for Notch1, such as DLL4 [57] and also TGF-β1 [58], which may be responsible for SLUG upregulation upon co-culture with HUVEC cells.

What could be the role of SLUG in gliomas? Here, we found that SLUG opposes GSC growth in vitro. This is in sharp contrast with previous results from Yang et al. [59], who found that SLUG promotes glioma growth and invasion. This discrepancy may have resulted from the use of the U251 cell line by Yang et al., which is cultured with serum and can genetically drift and lose the typical GBM profile with passages [60]. SLUG is involved in a wide variety of biological processes, such as tumor metastasis, epithelial-mesenchymal transition (EMT), stem cell biology, cellular differentiation, vascular remodeling, and DNA damage repair [61]. In addition, in the glioma context, both the Notch1 pathway [62,63] and SLUG gene expression have been found to be implicated in radio-resistance [64,65]. The link that we have established here between Notch1 activation and SLUG expression in GSC will serve as a basis to further understand how GBM cells resist radiotherapy and if SLUG is instrumental in this process.

Considering the roles of SLUG in vitro, it was necessary to demonstrate that SLUG is indeed expressed at the protein level in GBM patients. We found that SLUG was solely expressed in GBM, but not in grade II and grade III gliomas, by both tumor vascular cells and by tumoral cells, which confirms previous studies that highlighted its putative role in driving gliomagenesis and therapeutic resistance in GBM [24,25]. Our data indicate a conserved SLUG expression across all GBM samples we considered, accounting for an average 10% of total cells. In a similar study, SLUG was also expressed in tumor-associated pericytes, which were the major sources of this protein in GBMs [66]. Moreover, SLUG has been associated with poor prognosis and an aggressive mesenchymal GBM signature in genomic studies [24,25,67]. Interestingly, high numbers of tumor-associated pericytes expressing SLUG have recently been associated with increased neovascularization, malignancy, and therapeutic resistance in GBM, underscoring an important function of pericytes in gliomagenesis [68]. SLUG expression has been shown to be increased in vascular remodeling, where it appears to be associated with morphological changes and the proliferation of smooth muscle cells [68,69]. It is thus likely that the specific detection of SLUG observed in GBM vascular cells reflects its role in the vascular reorganization typically observed in high-grade gliomas.

Beside vascular cells, using a combined FISH-immunofluorescence technique, we identified a minor proportion of EGFR-amplified tumoral cells expressing SLUG in the two GBM patients we studied. SLUG is, thus, expressed both by the vascular microenvironment and tumoral cells. This dual tumoral vs. non tumoral expression of SLUG was also observed at the RNA level, using one recent glioblastoma RNA seq database (Figure S5E) [48]. Due to technical limitations, we did not fully demonstrate that these SLUG^+^ tumoral cells also co-expressed smooth muscle markers and adopted a pericyte-like phenotype as a result of a GBM-vascular trans-differentiation process [70,71,72]. More work using new multiplexing approaches would be necessary to address this issue and assess the contribution of SLUG^+^ GBM cells to GBM progression and, potentially, to neovascularization mechanisms. Using a mouse model, a TGF-β1–SLUG activation axis was recently shown to promote a pro-mesenchymal phenotype of glioma cells and a subsequent trans-differentiation towards a therapy-resistant state in vivo, thus demonstrating the functional involvement of SLUG as a driver of recurrence in GBMs [26]. The role in gliomagenesis and treatment resistance of SLUG^+^ cells in GBM patients remains to be explored. The specific isolation of these cells from patients would be very interesting, in order to study these properties in vitro.

Third, in addition to SLUG, we demonstrated that Notch1 activation induced the expression of the TAL1 protein in three GSC lines. The role of TAL1 in hematopoiesis, leukemia, and endothelial cell generation is well-known [40], yet this is the first demonstration of Notch1-induced expression of TAL1 in GSCs. Unexpectedly, only the truncated isoform TAL1-PP22 was induced by Notch1 in these cells. Most of our knowledge on TAL1 refers to the full length TAL1-PP42 isoform, whereas almost nothing is known about this short isoform. To date, its expression has only been observed during hematopoietic lineage specification [49,50,73,74] and results from complex transcriptional and post-transcriptional regulations [73,75]. TAL1-PP22 lacks a N-terminal proline-rich domain involved in transcriptional activation or repression [76,77]. Whether this truncated isoform exerts transcriptional activity in our GSCs is still unanswered.

All of our native GSC cultures express basal levels of LMO2, a well-documented transcriptional co-factor of TAL1 during hematopoiesis [78], erythropoiesis [31], and angiogenesis [39,79]. Following Notch1 activation, LMO2 is strongly upregulated in all GSCs and interacts with TAL1-PP22 in Gb4 and Gb7 cells. Previous studies have shown that LMO2 can promote GSC proliferation, invasion, and overall gliomagenesis [54,55]; a finding that is somehow opposite to our observations. Deciphering the precise function of the truncated TAL1-PP22 isoform will be key in fully understanding the molecular mechanisms involved in this process. It was proposed in other contexts that TAL1-PP22 functions as a trap, by titrating its partners and impairing their transcriptional functions [40]. Whether TAL1-PP22 exerts a similar function with LMO2 in GSCs requires further investigation. One could hypothesize that this “decoy” TAL1-PP22/LMO2 complex controls the GSC growth reduction observed downstream of NICD. In addition, one should not exclude the involvement of other known partners of TAL1, including GATA2, LMO4, and others [35,80], in our GSCs. Altogether, deeper mechanistic analyses are required to formally identify the respective transcriptional targets and modes of action of SLUG, TAL1-PP22, and LMO2 that mediate the growth reduction of GSCs and the associated phenotypical consequences.

Importantly, TAL1-PP22 was not only detected in vitro, but by also using protein extracts from glioma patient resections, indicating the potential relevance of this protein in gliomagenesis. In these glioma extracts, TAL1-PP22 was upregulated in GBMs, suggesting a specific role for this protein in high-grade glioma cells. As for SLUG, combined FISH-immunofluorescence showed that TAL1 is present in few GBM EGFR-amplified tumoral cells. Considering that TAL1 is involved in endothelial differentiation during development and controls active angiogenesis in later stages, it is tempting to speculate that these TAL1^+^ GBM cells are endothelial-like cells, which have been previously described [81,82] and would result from a trans-differentiation of GBM cells [70,71]. Additional work is needed to establish the identity of these TAL1^+^ GBM cells, their contribution to GBM progression, and if they potentially participate in neovascularization processes. The phenotype of TAL1^+^ cells in GBM resections, i.e., tumoral, endothelial, and microglial, was also supported by data from a GBM single cell RNA seq database (Figure S5F) [48]. Besides TAL1-PP22, the long TAL1-PP42 isoform was also observed in glioma extracts, both in low-grade and high-grade gliomas. Similar to SLUG, the expression of TAL1 is thus complex in gliomas, combining the expression of several isoforms in few glioma cells and in the tumor microenvironment. Whether these distinct isoforms exert distinct functions in this context remains to be elucidated.

## 5. Conclusions

We have uncovered the upregulation of both SLUG and the short isoform TAL1-PP22 downstream of Notch1 activation in GSCs. SLUG is also physiologically upregulated upon GSC differentiation, TGF-β1 treatment, and direct co-culture with endothelial cells, in some, but not all, GSC lines. In glioma patient samples, SLUG and truncated TAL1-PP22 are upregulated in GBMs in mutually exclusive subpopulations of cells. Using FISH, we demonstrated that these transcription factors are present in non-tumoral cells, but also, to a minor extent, in a fraction of EGFR-amplified tumoral cells. These results again reflect the cellular heterogeneity found in GBM and the complexity of the transcriptional circuitries regulating the diversity of GBM cell phenotypes. This work also raises new questions: what are the downstream transcriptional targets of SLUG and truncated TAL1? Besides reducing GSC growth, do they also control a vascular trans-differentiation as previously described in gliomas cells? Are they predictive markers of glioblastoma prognosis and response to treatments? Do they mediate therapeutic resistance in GBM and, thus, could we therapeutically target these transcription factors? Future work will address these important issues.

## Data Availability

The data related to this article are presented in the manuscript and Supplementary Materials.

## Supplementary Materials

The following figures and tables are available online at https://www.mdpi.com/article/10.3390/cancers13215393/s1. Figure S1. Antibody validation for immunofluorescence of SLUG and TAL1, Figure S2. SLUG and TAL1 expression upon Notch1 activation of GSCs, Figure S3. Gb4 cells upon modifications of culture conditions and co-cultures with HUVECs, Figure S4. TAL1 expression upon co-cultures of Gb4 cells with HUVECs, Figure S5. SLUG and TAL1 RNA profiling using human glioma genomic databases, Figure S6. EGFR amplification in GBM samples and presence of non-amplified SLUG^+^ and TAL1^+^ cells, Figure S7. SLUG and TAL1-PP22 lentiviral overexpression in GSCs, Figure S8. SLUG and TAL1 mRNA expression profiles in the Human Glioma Cell Culture biobank, Figure S9. Quantifications and statistics of western blot assays, Figure S10. Uncropped Figure 1C,F,G, Figure S11. Uncropped Figure 2C,F, Figure S12. Uncropped Figure 6B (blot 1–3), Figure S13. Uncropped Figure 7A, Figure S14. Uncropped Figure 7B (blot 1–3), Table S1. Detailed information of human samples used in the study, Table S2. Use of human samples in the study; Table S3. Detailed list of primary antibodies used in the study.
