# Supplementary material for: SLUG and Truncated TAL1 Reduce Glioblastoma Stem Cell Growth Downstream of Notch1 and Define Distinct Vascular Subpopulations in Glioblastoma Multiforme
Source: Cancers (Basel). 2021 Oct 27;13(21):5393. doi: 10.3390/cancers13215393 (PMC8582547; doi:10.3390/cancers13215393)
Supplement: Supplementary file 1 [file cancers-13-05393-s001.zip › cancers-1320808-supplementary.pdf]

Supplementary Material: SLUG and Truncated TAL1 Reduce Glioblastoma Stem Cell Growth Downstream of Notch1 and Define Distinct Vascular Subpopulations in Glioblastoma Multiforme

Sophie Guelfi *, Béatrice Orsetti, Virginie Deleuze, Valérie Rigau, Luc Bauchet, Hugues Duffau, Bernard Rothhut and Jean-Philippe Hugnot *

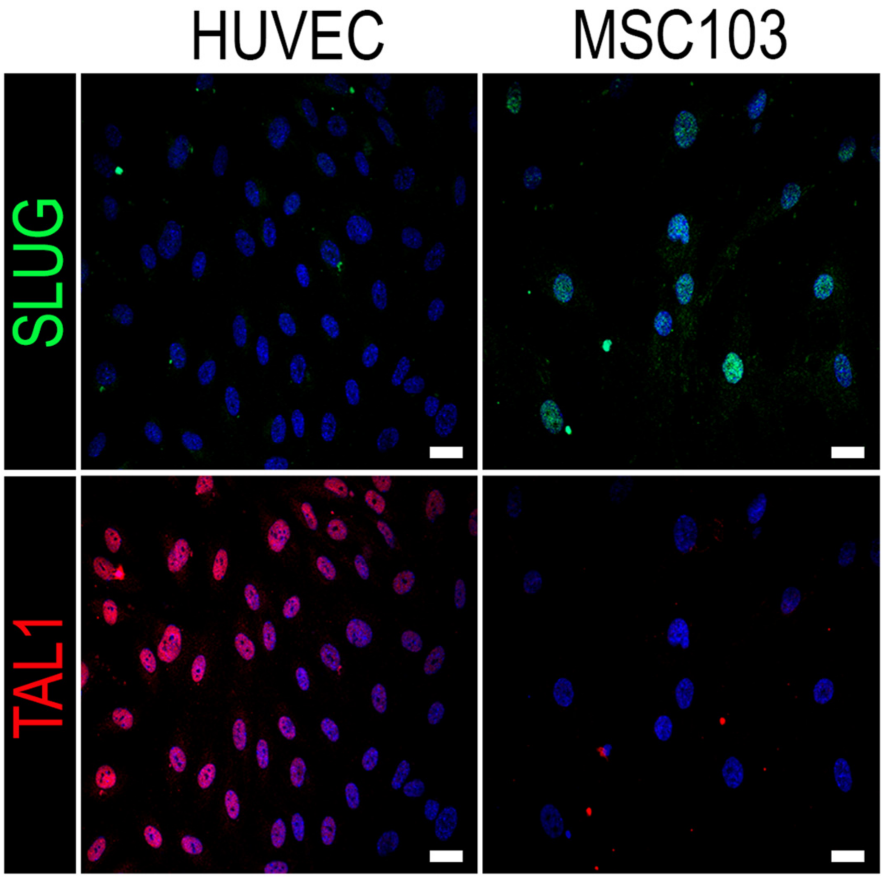

Figure S1. Antibody validation for immunofluorescence of SLUG and TAL1. Human primary mesenchymal stem cells MSC103 were used to validate the SLUG antibody used for immunofluorescence. HUVEC cells were used to validate the TAL1 antibody used for immunofluorescence. TAL1 is not expressed in MSC103 and conversely SLUG is not expressed in HUVECs. scales 20 µm. See Table S3 in the supplementary materials for descriptions of antibodies.

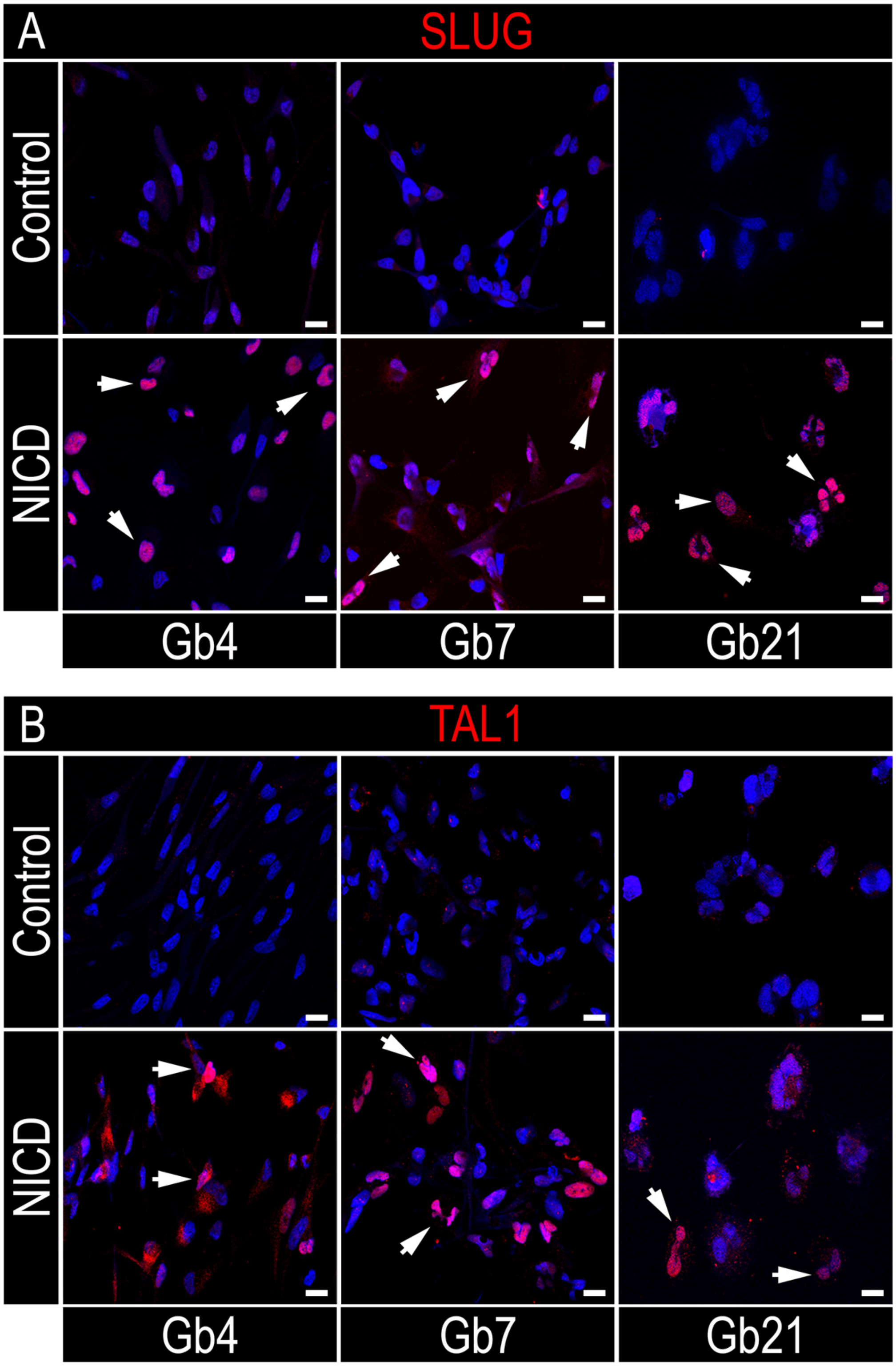

Figure S2. SLUG and TAL1 expression upon Notch1 activation of GSCs. Representative immunofluorescence (IF) images showing SLUG upregulation **(A)** and TAL1 induction **(B)** in Gb4, Gb7 and Gb21 cells upon the transduction of the Notch1 IntraCellular Domain (NICD) in proliferating adherent conditions (PDL/Laminin). Arrowheads indicate positive cells. scales 20 μm.

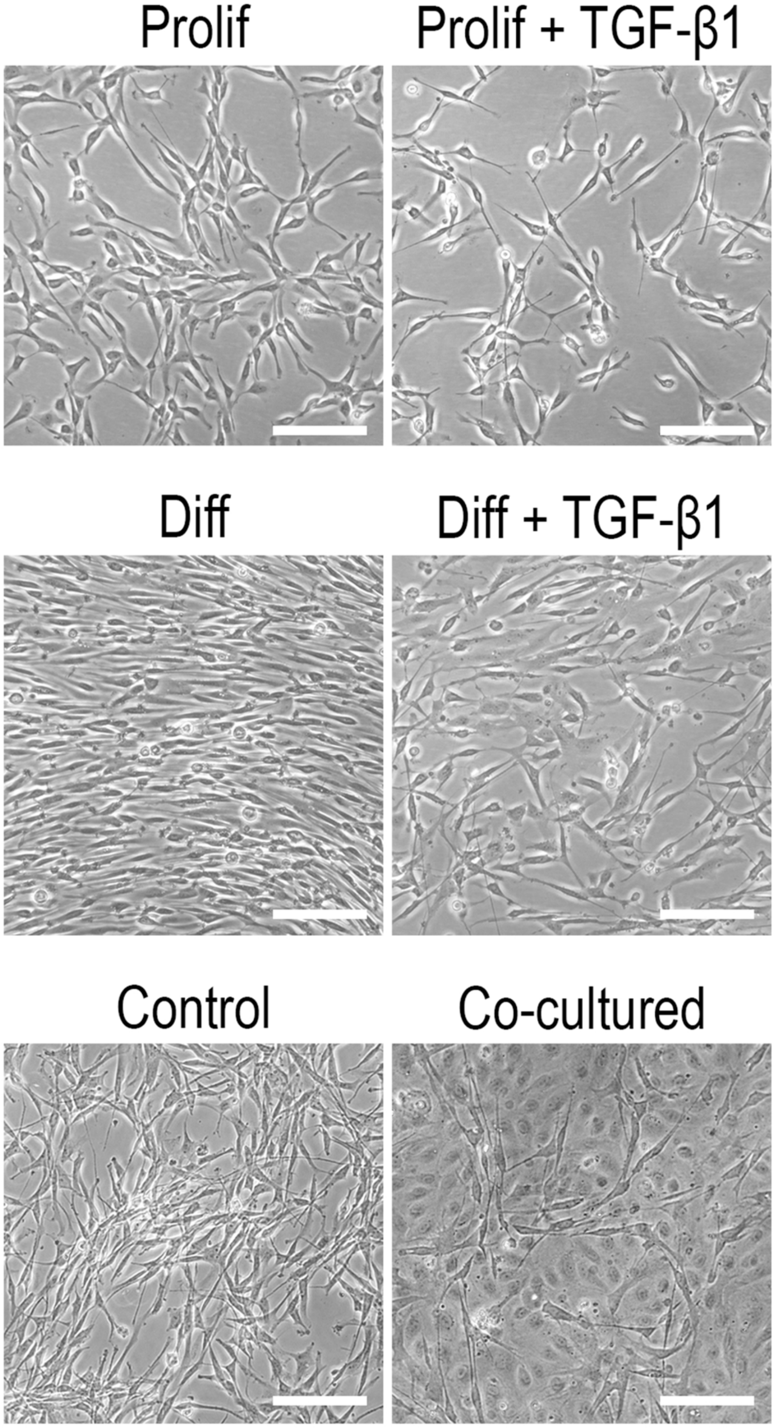

Figure S3. Gb4 cells upon modifications of culture conditions and co-cultures with HUVECs. Phase contrast images of Gb4 cells grown 5 days in proliferating adherent conditions (PDL/Lam) (upper panel), differentiating conditions (PDL/Lam) (middle panel), -/+ TGF-β1 treatment (2 ng/mL), and 72 h after coculture on top of HUVEC monolayers versus control gelatin conditions (lower panel). Morphological differences are observed upon differentiation and TGF-β1 treatment whereby the typical bipolar phenotype of Gb4 cells transitions towards a phenotype reminiscent of mesenchymal-like cells. This phenotype is partially observed in control conditions of cocultures (HUVEC media on gelatin) and increased in contact with HUVECs. scales 100 μm.

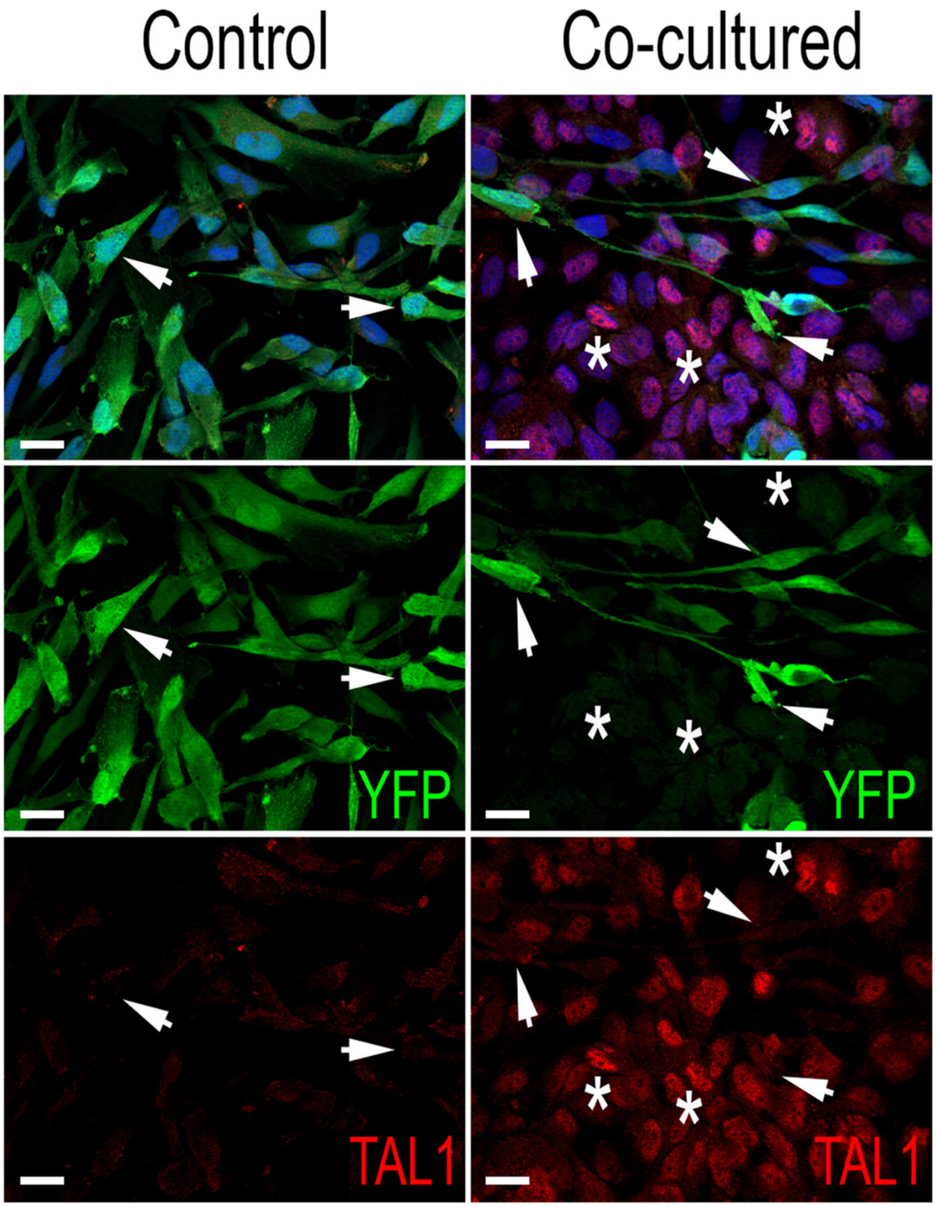

Figure S4. TAL1 expression upon co-cultures of Gb4 cells with HUVECs. Representative IF images of co-cultured Gb4 cells with HUVECs showing no induction of TAL1 in Gb4, but basal TAL1 expression in HUVECs. Arrowheads indicate YFP^{+}/TAL1^{-} Gb4 cells, stars indicate YFP^{-}/TAL1^{+} HUVEC cells. scales 20 μm.

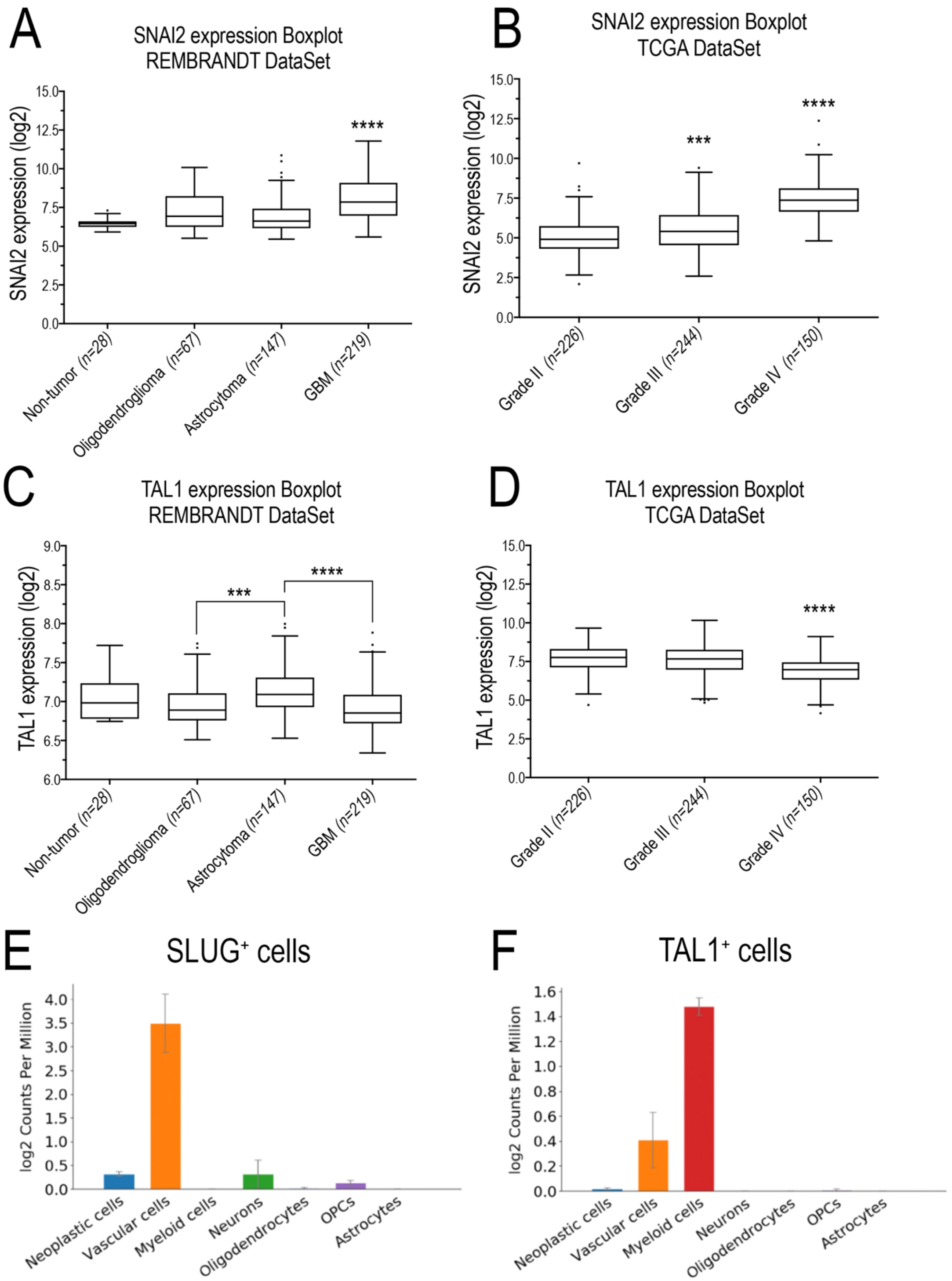

Figure S5: SLUG and TAL1 RNA profiling using human glioma genomic databases. (A,C) mRNA expression profiles for *SNAI2* (SLUG) **(A)** and *TAL1* **(C)** using the REMBRANDT database; and represented according to histological criterias used for glioma diagnosis. The dataset comprised a total of 461 human samples; including 28 samples of non-tumoral origin, 67 oligodendrogliomas, 147 astrocytomas and 219 GBMs. **(B,D)** mRNA expression profiles for *SNAI2* **(B)** and *TAL1* **(D)** using the TCGA-GBMLGG database, and represented according to glioma grade. The dataset comprised a total of 620 glioma samples; including 226 grade II samples, 244 grade III samples and 150 grade IV samples. Normalized datasets were downloaded from http://gliovis.bioinfo.cnio.es/ and replotted [1]. Expression intensities are shown as probeset values (log2). Plots are represented as Tukey's whisker boxes. Statistical analyses using one-way ANOVA tests with multiple comparisons between the mean of each group and Tukey's correction; ****, $p < 0.0001$ ***, $p < 0.001$. **(E,F)** Single cell RNA-seq analyses showing cell type expression of *SLUG* **(E)** and *TAL1* **(F)** across 4 human GBM samples and comprising 3589 cells. Plots available from www.gbmseq.org were directly integrated in the figure [2].

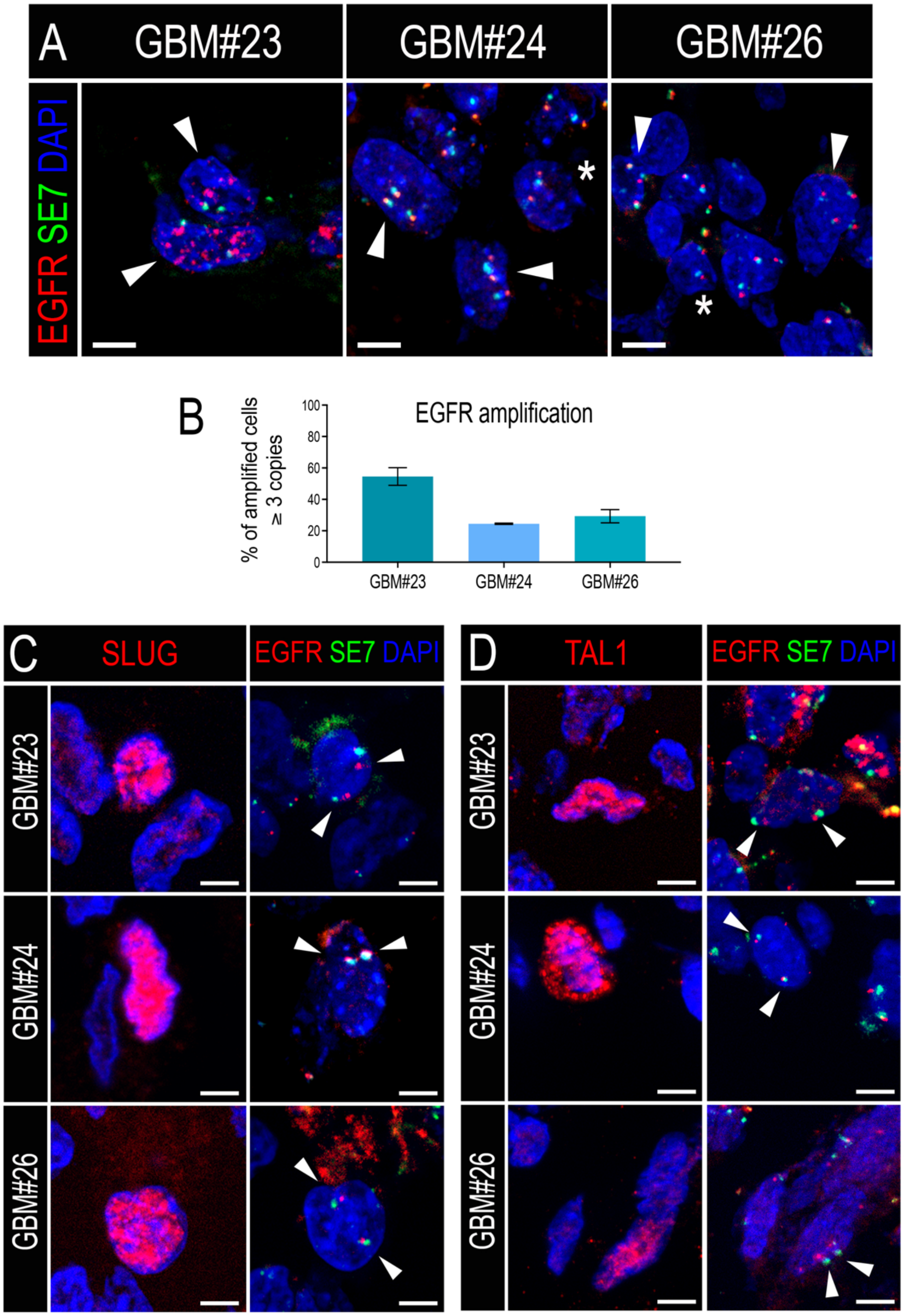

Figure S6: EGFR amplification in GBM samples and presence of non-amplified SLUG⁺ and TAL1⁺ cells. (A) Single *EGFR* locus analysis using dual EGFR/SE7 FISH probe hybridization in GBM#23, #24, #26. Arrowheads indicate amplified cells, stars indicate non-amplified cells if present in the same region of interest. **(B)** Quantification of *EGFR* amplification in GBM#23, #24 and #26. At least 200 cells were examined across multiple sections for each sample. Only cells with a clear *EGFR* locus signal (red dots), and a clear *SE7* signal (green dots) were considered for quantification. Cells with 3 *EGFR* copies or more were quantified as amplified, cells with 2 *EGFR* copies and 2 *SE7* copies were quantified as non-amplified. Amplified cells are represented as % of total considered cells for each sample. **(C,D)** Representative images of SLUG⁺ **(C)** and TAL1⁺ **(D)** cells not amplified for *EGFR* in GBM#23, #24 and #26. Left panels show IF for SLUG or TAL1 on selected cells, right panels indicate *EGFR*/*SE7* loci on the same cells following hybridization with the dual EGFR/SE7 FISH probe. Amplification was detected similar to **(A)** and Figure 5A,D. Arrowheads indicate *EGFR* loci in nuclei. Because of the applied sequential method and post-treatment for FISH hybridization, nuclei appear slightly different in right panels. DAPI: 4',6-diamidino-2-phenylindole; EGFR: *EGFR* locus-specific probe; SE7: satellite enumeration probe for chromosome 7. **(A,C,D)** scales 5 μm.

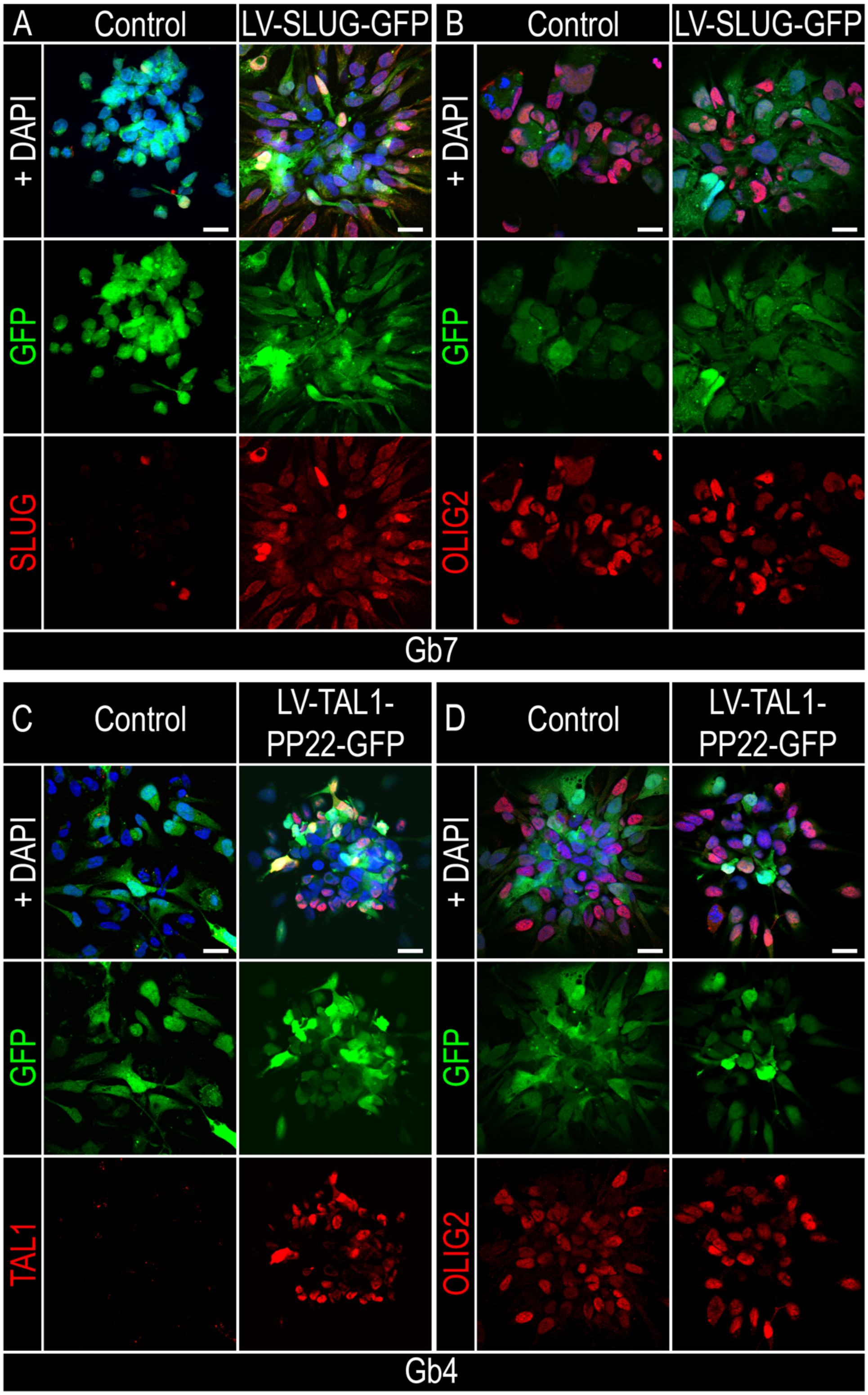

Figure S7. SLUG and TAL1-PP22 lentiviral overexpression in GSCs. Representative IF images of Gb4 and Gb7 5 days post-transduction using LV-SLUG-GFP **(A)** and LV-TAL1-PP22-GFP **(C)** lentiviruses, confirming a strong overexpression of these proteins, and showing no modulation of OLIG2 expression upon SLUG **(B)** or TAL1-PP22 overexpression **(D).** Cells were transduced in proliferating adherent conditions (PDL/Lam). scales 20 µm.

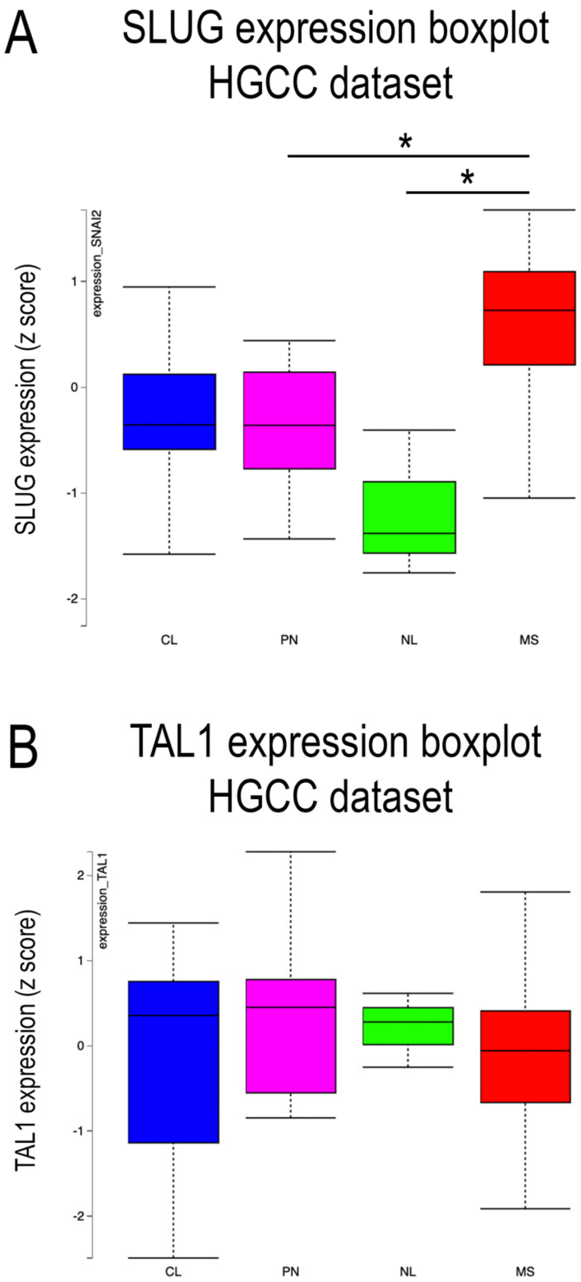

Figure S8. SLUG and TAL1 mRNA expression profiles in the Human Glioma Cell Culture (HGCC) biobank. **(A)** *SLUG (SNAI2)* mRNA expression profile in glioma cultures of different subtypes (HGCC biobank, CL = classical, PN = proneural, NL = neural, MS = mesenchymal) showing a significant upregulation of SLUG in mesenchymal subtype cells versus classical and proneural subtypes. **(B)** *TAL1* mRNA expression profile in HGGCs showing no significant upregulation of *TAL1* in specific subtypes of cells. Plots were used directly from the HGCC biobank, available at www.hggc.se (accessed on 6 January 2021), described in [3] and are represented as Tukey's whisker boxes. Datasets were downloaded for statistical analysis, comprising a total of 48 GBM cell lines, classified into 12 classical, 9 proneural, 3 neural, and 24 mesenchymal subtypes. Expression intensities are shown as z scores. Statistical analyses using one-way ANOVA tests with multiple comparisons between the mean of each group and Tukey's correction; *, $p \leq 0.05$. CL: classical; PN: proneural; NL: neural; MS: mesenchymal.

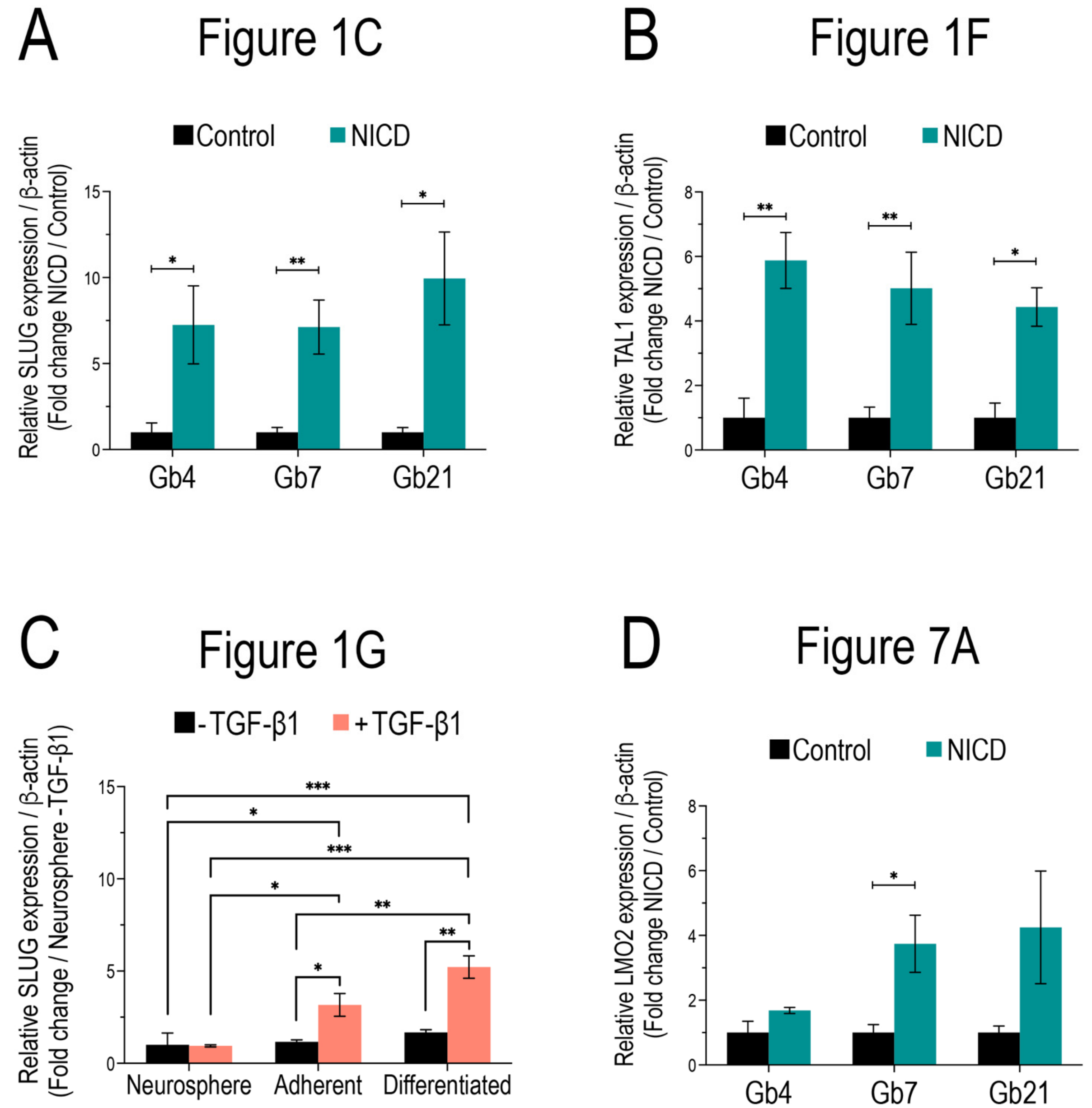

Figure S9: Quantifications and statistics of western blot assays. Quantifications and statistical analyses of western blot assays relative to Figure 1C **(A)**, 1F **(B)**, 1G **(C)** and Figure 7A **(D)**. Signal intensities of specific bands were quantified using ImageJ and normalized with β-actin bands as a loading control. For the expression of SLUG in Figure 1C **(A)**, TAL1 in Figure 1. F **(B)** and LMO2 in Figure 7A **(D)** following NICD transduction, band intensities are represented as fold changes normalized with control IRES-YFP conditions for each cell line. For the expression of SLUG in Figure 1G **(C)** following modifications of culture conditions and TGF-β1 treatment of Gb4, band intensities are represented as fold changes normalized with the neurosphere - TGF-β1 condition. Statistical analyses were performed using blot images of at least 3 independent experiments ($n = 3$); and consisted in unpaired student t tests for **(A,B,D)** and one-way ANOVA tests with multiple comparisons between the mean of each group and Tukey's correction for **(C)**. ***, $p < 0.001$; **, $p < 0.01$; *, $p \leq 0.05$.

Figure 1C

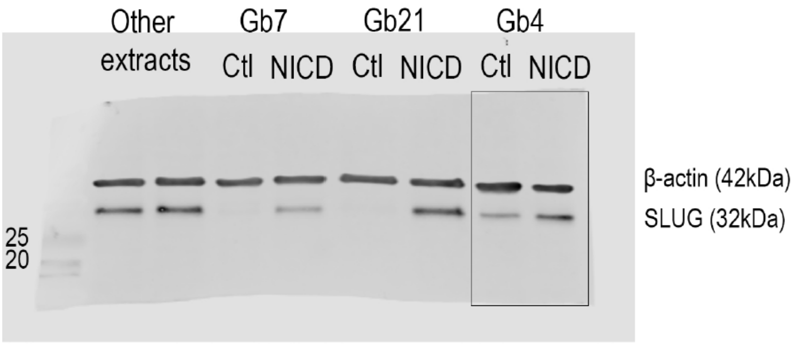

Gb4 lanes were moved on the left to fit with the reading order in the manuscript, it is clearly stated in the Figure1 legend. Ladder is visible at higher exposure times

Figure 1F

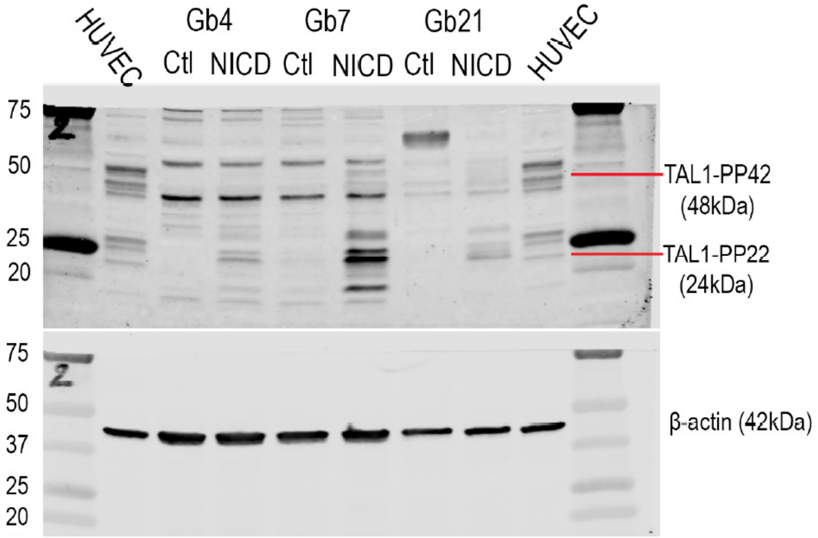

Figure 1G

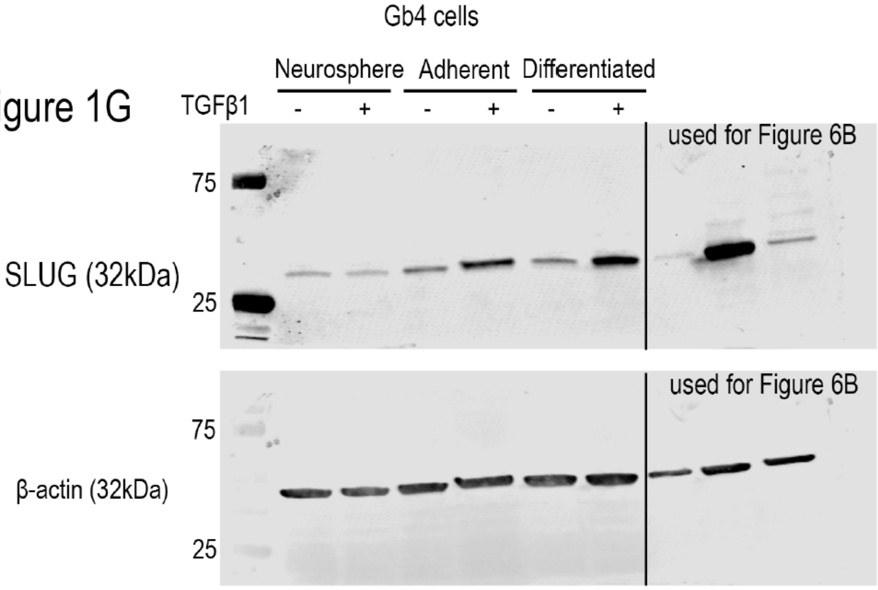

Figure S10. Uncropped Figure 1C,F,G.

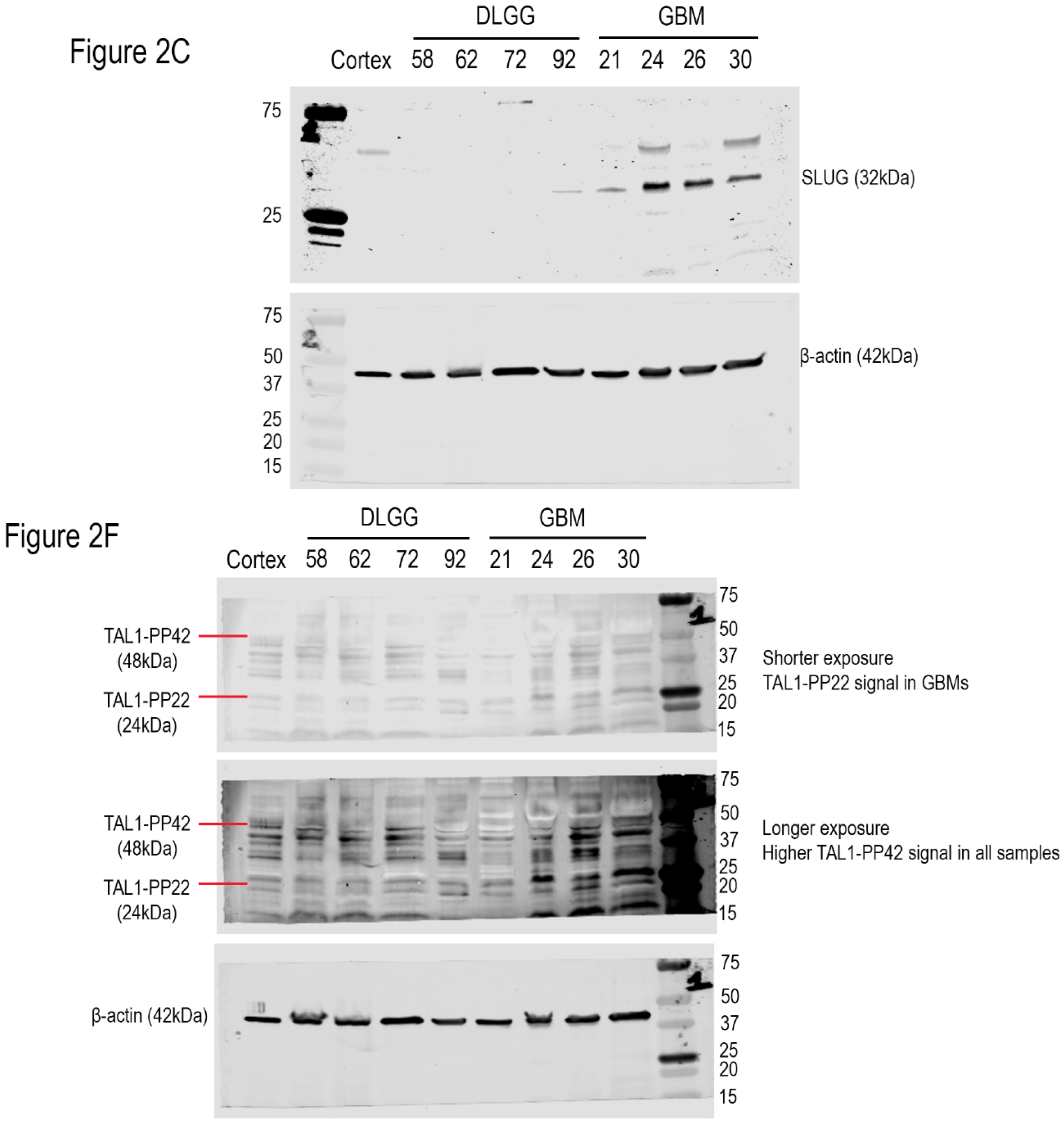

Figure S11. Uncropped Figure 2C,F.

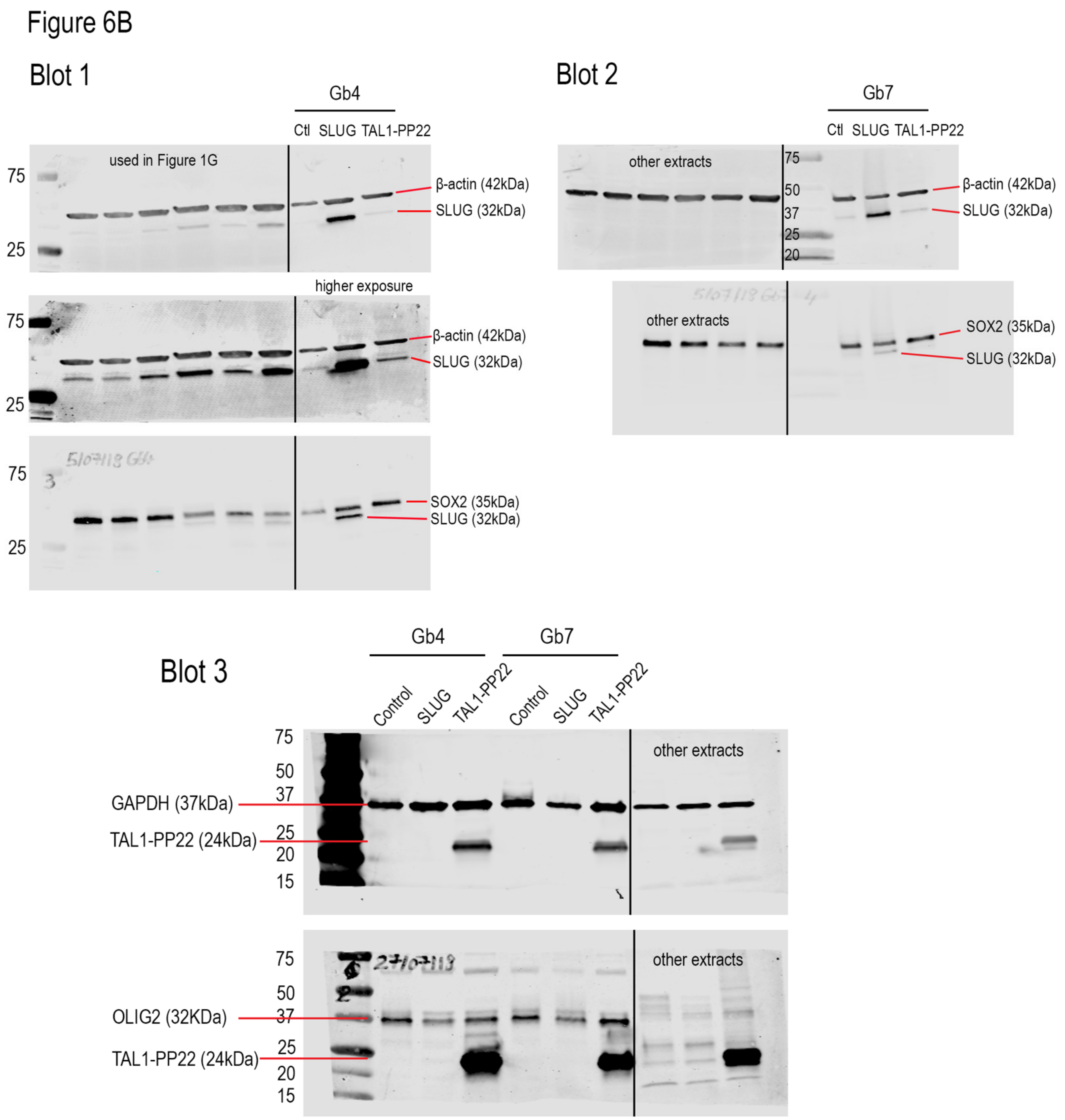

SLUG overexpression was controlled using Blot 1 for Gb4, Blot 2 for Gb7.
SOX2 expression upon overexpression of both SLUG and TAL1-PP22 versus control viruses was checked using Blot 1 (Gb4) and Blot 2 (Gb7) and quantitified relatively to the β-actin signal, with only slight differences in expression.
TAL1-PP22 overexpression was controlled using Blot 3 for both Gb4 and Gb7.
OLIG2 expression upon overexpression of both SLUG and TAL1-PP22 versus control viruses was checked using Blot 3 (Gb4 and Gb7) and quantitified relatively to the GAPDH signal, with only slight differences in expression.
Both loading control β-actin and GAPDH appear on the final figure, quantification method is explained in the figure legend.

Figure S12. Uncropped Figure 6B (blot 1–3).

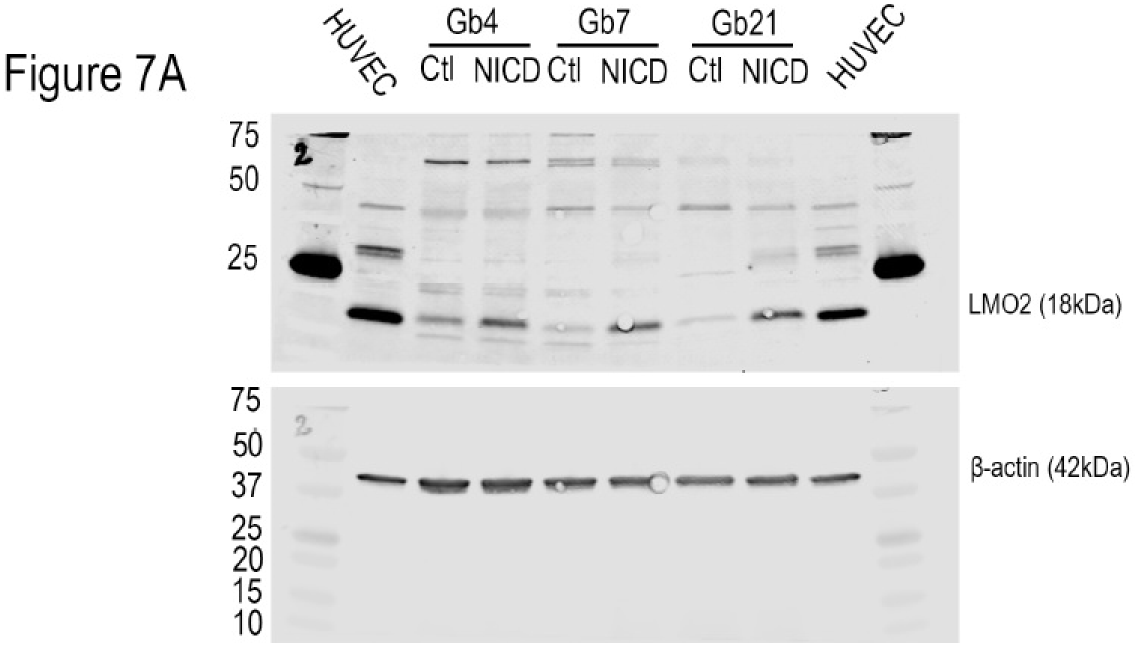

Figure S13. Uncropped Figure 7A.

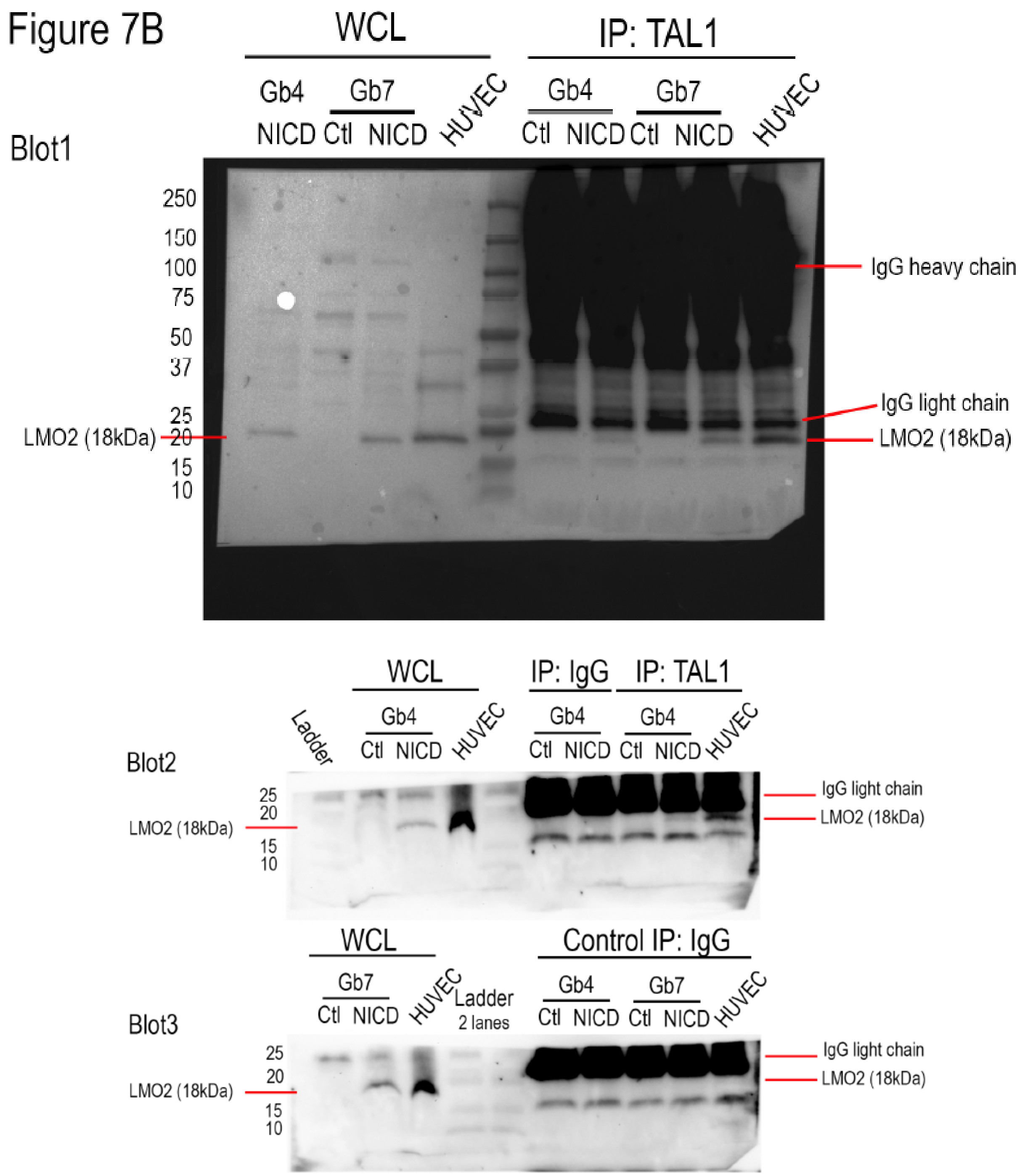

TAL1 Co-IP lysates of Gb4 and Gb7 upon NICD infection and HUVECs were loaded on the same gel (Blot 1) and were used for the final figure (right lanes from the ladder). Because of space limitation on the gel, control Co-IP with IgG lysates were loaded on a different gel (Blot 3) and were used for the final figure (right lanes from the ladder). Complete whole cell lysates of Gb4 upon NICD infection (Control+NICD) could not be loaded on Blot 1 with the Co-IP lysates because of space limitation and were loaded on Blot 2. On the final figure, the WCL Gb4 condition originates from Blot 2 while the WCL Gb7 + HUVEC condition originates from Blot1.

Figure S14. Uncropped Figure 7B (blot 1–3).

Table S1. Detailed information of human samples used in the study.

Tumor	Gender	Age	Localisation	P53 staining	Ki67 staining	EGFR staining	IDH1 mutation	1p19q deletion	ATRX	Diagnosis Subtype, Grade
**Cortex**	NA	NA	NA	NA	NA	NA	NA	NA	NA	NA
**DLGG#58**	M	26	Right frontal lobe	< 10%	1 - 10%	NA	IDH1 R132H	Deteted	Preserved	**Oligodendroglioma Grade II**
**DLGG#62**	M	26	Left motor cortex	> 60%	10 - 25%	NA	IDH1 R132H	Deteted	Loss	**Oligo or Astrocytoma Grade III**
**DLGG#72**	M	32	Left temporal lobe	30%	5%	NA	IDH1 R132H	No CoDel	Loss	**Astrocytoma Grade II**
**DLGG#89**	F	23	Left superior temporal gyrus	NA	NA	NA	IDH1 R132H	Partial loss of 19q	NA	**Oligodendroglioma Grade III**
**DLGG#92**	NA	NA	Parieto mesial	15%	NA	NA	IDH1 R132H	No CoDel	NA	**Oligo or Astrocytoma Grade II**
**GBM#21**	F	62	NA	80%	20%	NA	Not mutated	NA	NA	**Giant cell GB Grade IV**
**GBM#23**	F	77	Right Fronto-Insular	Negative	25%	Overexpressed	Not mutated	NA	NA	**GBM Grade IV**
**GBM#24**	F	39	Right Fronto-Insular	< 10%	30 - 40%	Overexpressed50%	Not mutated	NA	Preserved	**GBM Grade IV**
**GBM#26**	F	69	NA	40%	60%	Overexpressed	Not mutated	NA	NA	**Small cell GBM Grade IV**
**GBM#30**	M	65	NA	< 5%	30%	Overexpressed	Not mutated	NA	Preserved	**GBM Grade IV**

NA: not applicable

Table S2. Use of human samples in the study.

Tumor	Diagnosis Subtype, Grade	IHC	WB	IF SLUG + TAL1	Phenotype SLUG⁺ or TAL1⁺ cells	FISH + IF-FISH
**Cortex**	**NA**	**x**	**x**	NA	NA	NA
**DLGG#58**	**Oligodendroglioma Grade II**	**x**	**x**	NA	NA	NA
**DLGG#62**	**Oligo or Astrocytoma Grade III**	**x**	**x**	NA	NA	NA
**DLGG#72**	**Astrocytoma Grade II**	**x**	**x**	NA	NA	NA
**DLGG#89**	**Oligodendroglioma Grade III**	**x**	NA	NA	NA	NA
**DLGG#92**	**Oligo or Astrocytoma Grade II**	**x**	**x**	NA	NA	NA
**GBM#21**	**Giant cell GB Grade IV**	**x**	**x**	**x**	NA	NA
**GBM#23**	**GBM Grade IV**	**x**	NA	NA	NA	**x**
**GBM#24**	**GBM Grade IV**	**x**	**x**	**x**	**x**	**x**
**GBM#26**	**Small cell GBM Grade IV**	**x**	**x**	**x**	**x**	**x**
**GBM#30**	**GBM Grade IV**	**x**	**x**	**x**	NA	NA

X: used; NA: not applicable.

IF: immunofluorescence; IHC: immunohistochemistry; WB: western blot; FISH: fluorescence in situ hybridization; IF-FISH: sequential immunofluorescence followed by FISH analysis.

Table S3. Detailed list of primary antibodies used in the study.

Name	Species	Reference	IF/IHC Dilution	WB Dilution	Distributor	Use
TAL1	Goat	sc-12984	1:200	1:200	Santa Cruz	IF, WB, Co-IP
TAL1	Mouse	BTL73 clone	1:500	1:100	Gift from D. Mathieu	IF, IHC, WB
SLUG	Rabbit	9585	1:400	1:1000	Cell Signaling Technology	IF, IHC, WB
LMO2	Goat	AF2726	NA	1:1500	R&D Systems	WB
Olig2	Rabbit	18953	NA	1:400	IBL	WB
Sox2	Rabbit	23064	NA	1:400	Cell Signaling Technology	WB
CD31	Mouse	IR610	1:100	NA	Dako	IF
VE-Cadherin	Mouse	14-1449-80	1:100	NA	eBioscience	IF
αSMA	Mouse	M0851	1:1000	NA	Dako	IF
PDGFRβ	Rabbit	3169	1:250	NA	Cell Signaling Technology	IF
Iba1	Rabbit	019-19741	1:200	NA	Wako Chemicals	IF
GAPDH	Mouse	MAB374	NA	1:5000	Millipore	WB
ß-Actin	Mouse	3700	NA	1:5000	Cell Signaling Technology	WB

NA: not applicable.

Co-IP: co-immunoprecipitation; IF: immunofluorescence; IHC: immunohistochemistry; WB: western blot.

References

1. Bowman, R.L.; Wang, Q.; Carro, A.; Verhaak, R.G.; Squatrito, M. GlioVis data portal for visualization and analysis of brain tumor expression datasets. *Neuro-oncology* **2017**, *19*, 139-141, doi:10.1093/neuonc/now247.
2. Darmanis, S.; Sloan, S.A.; Croote, D.; Mignardi, M.; Chernikova, S.; Samghababi, P.; Zhang, Y.; Neff, N.; Kowarsky, M.; Caneda, C.; et al. Single-Cell RNA-Seq Analysis of Infiltrating Neoplastic Cells at the Migrating Front of Human Glioblastoma. *Cell Rep* **2017**, *21*, 1399-1410, doi:10.1016/j.celrep.2017.10.030.
3. Xie, Y.; Bergström, T.; Jiang, Y.; Johansson, P.; Marinescu, V.D.; Lindberg, N.; Segerman, A.; Wicher, G.; Niklasson, M.; Baskaran, S.; et al. The Human Glioblastoma Cell Culture Resource: Validated Cell Models Representing All Molecular Subtypes. *EBioMedicine* **2015**, *2*, 1351-1363, doi:10.1016/j.ebiom.2015.08.026.
